# Patterns of Alcohol Consumption and Associated Factors in a Population-Based Sample of 70-Year-Olds: Data from the Gothenburg H70 Birth Cohort Study 2014–16

**DOI:** 10.3390/ijerph19148248

**Published:** 2022-07-06

**Authors:** Felicia Ahlner, Hanna Falk Erhag, Lena Johansson, Madeleine Mellqvist Fässberg, Therese Rydberg Sterner, Jessica Samuelsson, Anna Zettergren, Margda Waern, Ingmar Skoog

**Affiliations:** 1 Neuropsychiatric Epidemiology Unit, Institute of Neuroscience and Physiology, Department of Psychiatry and Neurochemistry, Sahlgrenska Academy, Centre for Ageing and Health (AGECAP), University of Gothenburg, 43141 Mölndal, Sweden; hanna.falk@gu.se (H.F.E.); lena.maria.johansson@gu.se (L.J.); madeleine.mellqvist@gu.se (M.M.F.); therese.rydberg.sterner@gu.se (T.R.S.); jessica.samuelsson@gu.se (J.S.); anna.zettergren@gu.se (A.Z.); margda.waern@gu.se (M.W.); ingmar.skoog@gu.se (I.S.); 2 Aging Research Center, Department of Neurobiology, Care Sciences and Society, Karolinska Institutet, Stockholm University, 17165 Solna, Sweden; 3 Region Västra Götaland, Sahlgrenska University Hospital, Psychosis Clinic, 41345 Gothenburg, Sweden; 4 Region Västra Götaland, Sahlgrenska University Hospital, Psychiatry, Cognition and Old Age Psychiatry Clinic, 43141 Mölndal, Sweden

**Keywords:** alcohol use, determinants, characteristics, associated factors, older adults

## Abstract

Older adults of today consume more alcohol, yet knowledge about the factors associated with different consumption levels is limited in this age group. Based on the data from a population-based sample (*n* = 1156, 539 men and 617 women) in The Gothenburg H70 Birth Cohort Study 2014–16, we examined sociodemographic, social, and health-related factors associated with alcohol consumption levels in 70-year-olds, using logistic regression. Total weekly alcohol intake was calculated based on the self-reported amount of alcohol consumed. Alcohol consumption was categorized as lifetime abstention, former drinking, moderate consumption (≤98 g/week), and at-risk consumption (>98 g/week). At-risk consumption was further categorized into lower at-risk (98–196 g/week), medium at-risk (196–350 g/week), and higher at-risk (≥350 g/week). We found that among the 1156 participants, 3% were lifetime abstainers, 3% were former drinkers, 64% were moderate drinkers, and 30% were at-risk drinkers (20% lower, 8% medium, 2% higher). Among several factors, former drinking was associated with worse general self-rated health (OR 1.65, 95% CI 1.08–2.51) and lower health-related quality of life (measured by physical component score) (OR 0.94, 95% CI 0.91–0.97), higher illness burden (OR 1.16, 95% CI 1.07–1.27), and weaker grip strength (OR 0.96, 95% CI 0.94–0.98). Higher at-risk drinkers more often had liver disease (OR 11.41, 95% CI 3.48–37.37) and minor depression (OR 4.57, 95% CI 1.40–14.95), but less contacts with health care (OR 0.32, 95% CI 0.11–0.92). Our findings demonstrate the importance of classifications beyond abstinence and at-risk consumption, with implications for both the prevention and clinical management of unhealthy consumption patterns in older adults.

## 1. Introduction

Alcohol use is a major contributor to the global burden of disease [1], and a huge public health challenge [2]. Recent generations of older adults have higher consumption and alcohol misuse rates than previous generations [3,4]. The aging of the population worldwide may thus lead to a substantial increase in the absolute number of older adults who consume alcohol and alcohol-related injuries, diseases, and deaths [5].

The prevalence estimates of potentially harmful drinking among older adults vary considerably among studies, due to varying study contexts, drinking culture, and intake cut-offs. While there is a widespread consensus that men consume more alcohol and account for more alcohol-related harm to self and others at all ages [6], recent studies suggests a diminishing sex gap in alcohol use in later-born cohorts of older adults [3,7]. In Sweden, the proportion of current drinkers has been stable across age groups in recent years [8]. However, the higher consumption levels found among older adults aged 65–84 years are the opposite of the trends among the younger age groups, in which the consumption levels have decreased since 2004, with the most pronounced changes among those aged 17–29 years [8,9].

Despite the growing prevalence of alcohol use and misuse, harmful drinking often remains undetected and untreated among older adults [10]. Aging is related to slower alcohol metabolism [11], lean body-mass reduction [12], decreased water-to-body weight ratio [13], increased prevalence of health conditions, and adverse interaction with prescribed drugs [14], suggesting that older adults have an increased risk for adverse effects of alcohol consumption.

Older adults constitute a heterogeneous population, with considerable variations in physical, mental, and social health and well-being. In a recent review of qualitative studies, drinking in older age has been linked with social engagement, but also with social isolation, illness, or grief [15]. However, the findings are inconsistent regarding the broader characteristics of alcohol use and nonuse in older adults. Few studies have included a sufficiently large number of variables to capture the complexity of the factors associated with alcohol consumption levels in older age.

In alcohol research, the definitions of different levels of alcohol use and nonuse play a significant role. Nonuse includes both the lifetime abstainers and former drinkers. However, it is important to separate these two, since the former drinkers may differ from those who have abstained throughout life. The sick-quitter hypothesis, first suggested in the late 1980s, states that the former drinkers, to a greater extent, quit drinking due to serious health issues (including alcohol use disorders) or interaction with prescription drugs [16]. Despite this, few studies have separately analyzed the lifetime abstainers and former drinkers. There is evidence of heterogeneity in health and well-being among older adults with a high consumption [17]. Still, relatively little is known about the factors associated with high alcohol consumption levels in this age group. Given this gap in the literature, we aimed to explore a range of sociodemographic, social, and health-related factors in relation to the different categories of alcohol use and nonuse in a cross-sectional population-based sample of 70-year-olds born in 1944 and living in Gothenburg, Sweden.

## 2. Materials and Methods

### 2.1. Sample

The data were derived from the population-based Gothenburg H70 Birth Cohort studies (H70-studies), including systematically selected samples based on specific birth dates. In 2014–16, 1203 70-year-olds born in 1944 (response rate 72.2%) were examined by trained research staff with comprehensive social, somatic, cognitive, functional, and psychiatric examinations, and a battery of laboratory tests, as described previously [18]. The information was obtained through semi-structured face-to-face interviews, questionnaires, observations, and examinations. The H70 study was approved by the Regional Ethical Review Board (approval number: 869-13). Written informed consent was obtained from all of the participants. In the present study, 26 participants were excluded due to dementia, according to the Diagnostic and Statistical Manual of Mental Disorders (DSM-III-R) criteria [19], and eleven participants due to missing data on alcohol variables. Ten participants who reported that they stopped drinking within the past 0–5 years were also excluded to ensure a strict definition of alcohol abstention, leaving a total of 1156.

### 2.2. Measures

#### 2.2.1. Alcohol Variables

The information on alcohol consumption was obtained through semi-structured face-to-face interviews. The participants reported the average weekly amount and type of alcohol (beer, white wine, red wine, fortified wine, or liquor) consumed during the past month. Total weekly alcohol intake was calculated on the basis of the quantities reported. In accordance with the National Institute on Alcohol Abuse and Alcoholism (NIAAA) clinical guidelines for people aged 65 and above [20], the consumption levels among current drinkers were categorized as moderate (≤98 g/week) or at-risk (>98 g/week). In addition, at-risk drinkers were categorized into three distinct groups: lower at-risk drinkers (>98 to <196 g/week), medium at-risk drinkers (≥196 to <350 g/week), and higher at-risk drinkers (≥350 g/week). The nonusers were classified into two distinct groups: the lifetime abstainers (never-drinkers) or the former drinkers (previous drinkers, who had not consumed alcohol in the past five years).

#### 2.2.2. Independent Variables

All of the studied factors are presented in Table 1 and have been previously described in detail [18]. The sociodemographic and social factors were self-reported and included education, income, employment status, country of birth, housing, marital status, quality of marriage, having children and grandchildren, loss of partner due to death or divorce, loss of close relatives (children, grandchildren, siblings) or friends due to death, presence of a confidant relationship, satisfaction with social relationships, loneliness, childhood circumstances, religious beliefs, smoking status, frequency of physical activity, and satisfaction with sleep. The potential consequences of current or previous alcohol use were assessed by question 9 (‘Have you or someone else been injured as a result of your drinking?’) and question 10 (‘Has a relative or friend or doctor or another health worker been concerned about your drinking or suggested you to cut down?’) from the Alcohol Use Disorders Identification Test (AUDIT) [21].

The detailed information on instrument, scales, and the system used for measurement of health status is found in Table 2. The health status measures included self-reports, performance tests, and validated instruments and scales. The self-reported measurements included information on general health, life satisfaction, the number of medications, the prevalence of diagnosed medical conditions (stroke, hypertension, angina pectoris, myocardial infarction, diabetes mellitus, liver disease, lifetime occurrence of all cancer types), and injuries (hip or femoral fractures, falls, head trauma), need of home health care, hospitalization, and other contacts with medical doctor or nurse.

The physical performance measures included grip strength, gait speed (30 m indoor at a self-selected pace with a standing start), and vital capacity (forced expiratory volume in one second divided by forced vital capacity). Body mass index (BMI) was calculated (body weight divided by height squared). Unhealthy weight was defined as BMI ≥31, based on the suggested age-adjusted cut-off for people aged 65 and over [22].

The 36-Item Short Form Health Survey (SF-36) [23] was used for a summary of health-related quality of life (HRQoL). Standard procedures were used for the coding of electrocardiographic findings. Functional independence, the burden of illness, cognitive function, and symptoms of anxiety and personality were rated with established instruments and tests. Diagnoses of depression (i.e., minor and major) were based on computerized symptom algorithms, according to criteria in the Diagnostic and Statistical Manual of Mental Disorders (DSM-IV-TR, DSM-5) [24,25].

**Table 2:** Detailed information on instrument, scales and system used for measurement of health status.

	Measure	Instrument, Scale or System Used for Classification	Reference
Physical health	Self-reported general health	The 36-Item Short Form Health Survey (SF-36)	[23]
	Physical Component Score	The 36-Item Short Form Health Survey (SF-36)	[26]
	Electrocardiography	The Minnesota Code Classification System	[27]
Functional independence	Activities of Daily Living (ADL)	The Barthel ADL Index	[28]
	Instrumental activities of daily living (IADL)	The Lawton IADL Scale	[29]
Burden of disease	Burden of illness	Cumulative Illness Rating Scale for Geriatrics (CIRS-G)	[30]
Mental health	Cognitive function	Mini Mental State Examination (MMSE)	[31]
	Anxiety	The Brief Scale for Anxiety (BSA)	[32]
	Depressive symptoms	The Montgomery Åsberg Depression Rating Scale (MADRS)	[33]
	Mental Component Score	The 36-Item Short Form Health Survey (SF-36)	[26]
	Minor depression disorder	The Diagnostic and Statistical Manual of Mental Disorders, 4th ed. (DSM-IV-TR)	[24]
	Major depression disorder	The Diagnostic and Statistical Manual of Mental Disorders, 5th ed. (DSM-5)	[25]
Personality	Extroversion	The Eysenck Personality Inventory	[34]
	Neuroticism	The Eysenck Personality Inventory	[34]

### 2.3. Statistical Analysis

The sample characteristics are presented as numbers, median values, minimum (min) and maximum (max) values, and percentages. Pearson’s chi-square test was used to test differences in proportions. The Mann–Whitney U test and the Kruskal–Wallis test were used to test differences in medians. Logistic regression models were used to test associations between sociodemographic/social/health-related factors (independent variables) and alcohol consumption levels (outcome). A total of 56 factors were examined. Due to the exploratory nature of the present study, the data were analyzed without multiplicity adjustment. Statistically significant associations are presented in tables containing odds ratios (OR) adjusted for sex (Model 1) with 95% confidence intervals (CI) and *p*-values. The complete tables with all of the independent variables, including unadjusted OR and OR adjusted for sex and education (Model 2), can be found in Tables S1–S3, Supplementary Materials. All of the analyses were carried out using IBM SPSS Statistics 24 for Windows. A *p*-value < 0.05 (two-tailed) was considered statistically significant. Significant associations were further examined for potential interactions with sex to assess the need for stratified models. The interaction *p*-value threshold was ≤0.1.

## 3. Results

### 3.1. Sample Characteristics

The mean age of the participants (*n* = 1156) was 70.6 years, and 53.4% were women. Table 3 shows that, compared to men, women had a lower educational level, lower income, were less often in a relationship, more often lived alone, and were more often religious. Out of 1156 participants, 64 (5.5%) were nonusers, out of which 32 (2.8%) were lifetime abstainers and 32 (2.8%) were former drinkers. A total of 1092 (94.5%) were current drinkers, out of which 745 (64.4%) were moderate drinkers and 347 (30.0%) were at-risk drinkers. Among the at-risk drinkers, 229 (19.8%) were lower at-risk, 92 (8.0%) were medium at-risk, and 26 (2.2%) were higher at-risk drinkers. The median consumption was 32 g of alcohol per week among the moderate drinkers, and 160 g per week among the at-risk drinkers; 135 g among the lower at-risk, 249 g among the medium at-risk, and 481 g among the higher at-risk drinkers. Among those with at-risk consumption, men were more likely than women to be medium/higher at-risk drinkers, x2 (2, *n* = 347) = 7.19, *p* = 0.03.

### 3.2. Factors Associated with Different Alcohol Consumption Levels

Table 4, Table 5 and Table 6 show the statistically significant results of the logistic regression models adjusted for sex (Model 1). The complete result tables for Model 1 and Model 2 are presented in Tables S1–S3, Supplementary Materials.

Compared to the moderate drinkers, the lifetime abstainers had a lower income, had less often lost relatives during the last five years, were more often religious, never-smokers, and physically inactive, had less often contact with health care, but had more often minor depression (Table 4). The associations remained similar in Model 2 (Table S1, Supplementary Materials). An interaction effect was observed between sex and physical inactivity (*p* = 0.008), indicating that the association between physical inactivity and lifetime abstention was different in men and women. Stratified analysis showed that physical inactivity was associated with lifetime abstention among women (OR 5.95, 95% CI 1.98–17.87, *p* = 0.001), but not among men (OR 0.56, 95% CI 0.14–2.20, *p* = 0.40).

Compared to the moderate drinkers, the former drinkers had a lower income, were less often born in Sweden, had more often experienced an unhappy childhood and alcohol-related injuries, were more often smokers, physically inactive, and dissatisfied with sleep, had lower life satisfaction, and had poorer general self-rated health, a worse HRQoL in the physical component score (PCS), a higher burden of illness (CIRS-G), had more medications, more often had hypertension, were more frequently hospitalized, had more hospital admitted head injuries, weaker grip strength, slower gait speed, had unhealthy weight, a higher MADRS score, and more often had minor depression (Table 4). The associations remained similar in Model 2 (Table S1, Supplementary Materials). No interactions with sex were observed.

Compared to the moderate drinkers, the at-risk drinkers had higher education, a higher income, were more often born in Sweden, more often in a relationship, and had more often experienced partner loss (>5 years). They also showed a higher financial standard during childhood, were more often smokers, less often religious, more often acknowledged that others were concerned about their drinking, had diabetes less often, and had a higher MMSE score (Table 4). With the exception of the MMSE score, the associations remained similar in Model 2 (Table S1, Supplementary Materials). No interactions with sex were observed. Compared to the moderate drinkers, the lower at-risk drinkers had higher education, a higher income, were more often born in Sweden and in a relationship, were less likely to live alone, had more often experienced partner loss (>5 years), had a good financial standard during childhood, were more often smokers, less often religious, more often acknowledged that others were concerned about their drinking, had higher life satisfaction, had myocardial infarction and diabetes less often, had better grip strength, and had higher MMSE scores (Table 5). The associations, except for the MMSE score, remained similar in Model 2 (Table S2, Supplementary Materials). Interactions were observed between sex and smoking (*p* = 0.06) and sex and grip strength (*p* = 0.08), indicating that the associations were different in men and women. The stratified analyses showed that smoking was associated with the lower at-risk drinking among women (OR 2.31, 95% CI 1.35–3.93, *p* = 0.002) but not among men (OR 1.20, 95% CI 0.79–1.81, *p* = 0.395). A better grip strength was associated with the lower at-risk drinking among men (OR 1.02, 95% CI 1.01–1.04, *p* = 0.002), but not among women (OR 1.00, 95% CI 0.99–1.02, *p* = 0.836).

Compared to the moderate drinkers, the medium at-risk drinkers had higher education, a higher income, were more often born in Sweden, were more often smokers, less often religious, had more often acknowledged that others were concerned about their drinking, and had a higher burden of illness (CIRS-G) (Table 5). The associations, except for the MMSE score, remained similar in Model 2 (Table S2, Supplementary Materials). An interaction effect was observed between sex and smoking (*p* = 0.09), indicating that the association between smoking and medium at-risk drinking was different in men and women. Stratified analyses showed that smoking was related to the medium at-risk drinking among men (OR 3.17, 95% CI 1.63–6.19, *p* = 0.001), but not among women (OR 1.24, 95% CI 0.54–2.87, *p* = 0.612).

Compared to the moderate drinkers, the higher at-risk drinkers had more often had a higher financial standard during childhood and had more often acknowledged that others were concerned about their drinking, had less contact with healthcare, a higher burden of illness (CIRS-G), liver disease, and minor depression (Table 5). The associations remained similar in Model 2 (Table S2, Supplementary Materials). No interactions with sex were observed.

We then compared the two nonuser groups. Compared to the lifetime abstainers, the former drinkers had more often experienced partner loss (>5 years) and an unhappy childhood, were more often smokers and dissatisfied with sleep, had a worse HRQoL in the physical component score (PCS), a higher burden of illness (CIRS-G), had more medications, were more often hospitalized, had more hospital admissions for head injuries, weaker grip strength, and slower gait speed (Table 6). The associations remained similar in Model 2 (Table S3, Supplementary Materials). No interactions with sex were observed.

We then compared the former drinkers with the at-risk drinkers. Compared to the at-risk drinkers, the former drinkers had lower education, lower income, were less often born in Sweden, less often in a relationship, were more likely to live alone, had a worse financial standard during childhood, more often had an unhappy childhood, were more often religious, physically inactive, dissatisfied with sleep, had a lower life satisfaction, poorer general self-rated health, worse HRQoL in the physical component score (PCS), were more functionally dependent (ADL), had a higher burden of illness (CIRS-G), had more medications, had angina pectoris and diabetes more often, had been hospitalized more often, had more hospital-admitted head injuries, had a weaker grip strength, and slower gait speed (Table 6). The associations, except for the financial standard during childhood and physical inactivity, remained similar in Model 2 (Table S3, Supplementary Materials). Interactions were observed between sex and higher education level (*p* = 0.03), sex and financial standard during childhood (*p* = 0.07), sex and sleep satisfaction (*p* = 0.02), and sex and grip strength (*p* = 0.07), indicating that the associations were different in men and women. The stratified analysis showed that higher education and a worse financial standard were associated with former drinking among women (education: OR 0.06, 95% CI 0.01–0.31, *p* = 0.001; financial standard: OR 6.38, 95% CI 1.40–29.01, *p* = 0.02), but not among men (education: OR 0.63, 95% CI 0.16–2.52, *p* = 0.51; financial standard: OR 1.14, 95% CI 0.37–3.51, *p* = 0.82). Sleep dissatisfaction and weaker grip strength were associated with former drinking among men (sleep dissatisfaction: OR 17.70, 95% CI 4.70–66.72, *p* < 0.001; grip strength: OR 0.92, 95% CI 0.88–0.96, *p* < 0.001), but not among women (sleep dissatisfaction: OR 1.68, 95% CI 0.43–6.64, *p* = 0.46; grip strength: OR 0.97, 95% CI 0.93–1.02, *p* = 0.22).

## 4. Discussion

We examined a wide range of the sociodemographic, social, and health-related factors associated with alcohol consumption levels in a population-based sample of 70-year-olds. We found that the former drinkers, and, to a certain extent, the higher at-risk drinkers, had poorer health, while there were few differences in health between the moderate drinkers and the at-risk drinkers. There were also sociodemographic and social differences between the groups. The former drinkers were more often smokers and born outside Sweden, while the at-risk drinkers had higher education, higher income, and were more often in a relationship. Our findings show the importance of differentiating between the lifetime abstainers and the former drinkers, and of differentiating between the different consumption levels among the at-risk consumers, as these subgroups differ considerably in relation to sociodemographic, social, and health-related factors.

Nearly one-third (30.0%) of Swedish 70-year-olds born in 1944 in our study exceeded the NIAAA drinking guidelines (>98 g/week), a figure which is higher than in previous studies on adults aged 65 years and over [35,36,37]. A Belgian study reported a prevalence of 20.5% [35], while a survey of older Medicare beneficiaries in the US reported a 13.0% prevalence [36], and among US primary care patients the prevalence was 7.9% [37]. The larger proportion of the higher at-risk drinkers (≥350 g/week) in our study (2.2%) was similar to that reported from a population-based study among people aged 65 and over in Japan (1%) [38], but lower than the results from a European consortium study including all ages (mean age 57 years: 7.5%) [39]. However, comparisons between the studies are difficult, due to large variations in terms of the age groups, divergent definitions of alcohol consumption groups, and cut-offs.

In our study, the former drinkers had the worst physical and medical status. This finding is in line with the sick-quitter hypothesis [16]. We found few differences between the at-risk drinkers and the moderate drinkers regarding health-related factors, even after controlling for education. However, the higher at-risk drinkers (≥350 g/week) had more liver disease and minor depression, established consequences of alcohol dependence and excessive drinking at various ages and in different settings [40,41], but rarely studied in older populations. The moderate drinkers had diabetes more often, which might be explained by the general recommendation to diabetics to drink in moderation.

We found that minor depression was associated with both nonuse (lifetime abstention and former drinking) and the higher at-risk drinking, indicating a curvilinear association, as previously reported among older adults [42,43,44]. One reason for the higher rate of depression among the abstainers may be the social aspects of drinking, in which depressed persons are less likely to be engaged [45]. However, the cross-sectional design of this study makes it impossible to speculate about the direction of this relationship. Among the higher at-risk drinkers, alcohol might be a self-medicating strategy to manage the symptoms of minor depression [46]. However, the research findings are inconsistent regarding alcohol consumption and late-life depression, and several studies find no associations [47,48,49]. Depression is related to lower life satisfaction [50]. In our study, lower life satisfaction was associated with former drinking, but no association was found with high consumption levels. Our findings are similar to a Russian population study (mean age 43.2), which reported a hump-shaped relationship (J-shaped for men, and U-shaped for women) between alcohol consumption and life satisfaction [51], but in contrast to the findings among older Jamaicans (aged 60–103 years), where current drinking was associated with both very high and very low life satisfaction [52].

Most of the differences between the lifetime abstainers and current drinkers (moderate and at-risk) involved sociodemographic characteristics. We found that lifetime abstention was associated with lower education, lower income, non-smoking, and being religious, which is similar to a study among people aged 65 and over in the US [53]. Higher consumption was related to higher education and income, and being a smoker, as also found among the Medicare beneficiaries aged 65 and over in the US [36]. We found that those with a partner were more likely to be at-risk drinkers, which is in line with findings among older adults (≥65 years) in Finland [54]. However, these findings are in contrast to studies from the US and Japan (age: ≥65 years), where being single or divorced was associated with a higher likelihood of unhealthy drinking [36,38]. These results suggest that the influence of marital status on alcohol consumption is complex and varies between geographical areas and over the life course. Additionally, the associations between alcohol consumption and the country of birth and religiousness further point to the importance of cultural influences. New findings from our study regarding sociodemographic factors include that at-risk drinking was associated with partner loss, others’ concern about the drinking, and a good financial standard during childhood.

We found that nonusers (lifetime abstainers and former drinkers) were less physically active than current drinkers, which is similar to studies among college students and in the general population [55]. Sex as a potential moderator in the relationship between physical activity and alcohol use was previously observed in a US sample of adults aged 18 and above [56]. However, that study found a stronger association in men, while the association was only apparent among women in our study, indicating an effect of age.

Overall, recent pre-clinical research has found that excessive alcohol exposure during adolescence may increase the risk of chronic health issues, especially in terms of brain damage [57,58]. Unfortunately, we have no information about the consumption pattern during adolescence. Thus, we cannot investigate whether previous alcohol habits have influenced health and function in our sample.

### Strengths and Limitations

The strengths of our study include the systematically selected population-based sample, the comprehensive examinations, and the high response rate. In addition, we provide separate data for the lifetime abstainers and former drinkers. The study also has several limitations. First, the cross-sectional design limits the possibility of making causal inferences and cannot address the long-term effects of elevated drinking in old age. Second, when using self-reported alcohol consumption data, there is a risk that some individuals may underestimate or under-report their consumption to provide a more socially desirable response. However, self-reported alcohol consumption is considered to be reliable and valid [59]. To limit recall bias, participants reported the past month consumption. Underestimation was further minimized by using questions about the frequency and amounts consumed for beer, wine, and spirits separately, which yields the most realistic levels of intake [60]. However, due to the potential monthly variation in alcohol use, the reported past month consumption may not reflect the actual annual consumption pattern. Third, some medical conditions are based on self-report and were not confirmed by clinical examinations or registers. Fourth, some of the subgroups were small, leading to low statistical power and risk of false negative results (Type II errors), large risk estimates, wide confidence intervals, and the difficulties in controlling for all of the potential confounders. Although we found some interactions by sex, the absence of sex differences for other variables may be due to the small sample sizes [61]. However, the latter risk was reduced by the use of a higher *p*-value threshold (*p* < 0.1) for the interaction analyses. Fifth, this is an exploratory study and multiple comparisons were made, which may lead to false positive findings (Type I errors). However, multiplicity adjustments may increase the risk of false negative findings (Type II errors). Instead, all of the comparisons are clearly presented with individual *p*-values and confidence intervals. This approach enables the reader to interpret the results and decide whether the results are entirely due to multiple comparisons. Moreover, we emphasize that our findings are suggestive until further confirmed. However, we take note of the fact that the associations found are plausible and explicable [62,63]. Finally, as in all studies involving older adults, survival bias may also explain our findings, as long-term heavy users might have died before the age of 70, or might have stopped drinking at an earlier age.

## 5. Conclusions

In conclusion, we found that unfavorable factors were more common among the former drinkers and the individuals in the highest consumption groups. Our study underlines the wide variation in the factors associated with levels of alcohol use among older adults. Our findings suggest that there are important similarities and discrepancies within the subgroups at the extremes of alcohol use (abstainers and high consumers). These findings demonstrate the importance of accurate classification of alcohol consumption groups beyond abstinence and risk consumption in future research, as these definitions have importance for the outcome and interpretation of results. Our findings shed new light on understanding older alcohol consumers, which may help in improving prevention and treatment of individuals with unhealthy consumption patterns.

## Figures and Tables

**Table 1:** Categorization of included variables; type, values, and collection method.

Variable	Type	Value	Variable	Type	Value
**Sociodemographic factors**			**Physical health cont.**		
Education ^a^	Categorical	≤Primary *, Secondary, Higher	Medications ^a^	Continuous	Number of medications
Income ^a^	Categorical	<Sample median, ≥Sample median *	Stroke ^a,^*	Categorical	No *, Yes
Employed ^a^	Categorical	No *, Yes	Hypertension ^a,^*	Categorical	No *, Yes
Born in Sweden ^a^	Categorical	No, Yes *	Angina pectoris ^a,^*	Categorical	No *, Yes
Special housing ^b^	Categorical	No *, Yes	Myocardial infarction ^a,^*	Categorical	No *, Yes
Having partner ^a^	Categorical	No *, Yes	Diabetes ^a,^*	Categorical	No *, Yes
Happy relationship ^a^	Categorical	No *, Yes	Liver disease ^a,^*	Categorical	No *, Yes
Living alone ^a^	Categorical	No *, Yes	Cancer (any) ^a,^*	Categorical	No *, Yes
Having children ^a^	Categorical	No *, Yes	Hip or femoral fracture preceding 10 years ^a^	Categorical	No *, Yes
Having grandchildren ^a^	Categorical	No *, Yes	Have fallen preceding 1 year ^a^	Categorical	No *, Yes
Loss of partner due to death or divorce ^a^	Categorical	No *, Yes, more than 5 years ago, Yes, 0–5 years ago	Home care ≥1/week ^a^	Categorical	No *, Yes
Lost relatives or friends due to death ^a^	Categorical	No *, Yes, more than 5 years ago, Yes, 0–5 years ago	Hospitalization preceding 10 years ^a^	Categorical	No *, Yes
Having ≥ 1 confidant ^a^	Categorical	No, Yes *	Hospital admitted head injury ^a^	Categorical	No *, Yes
Feeling alone ^a^	Categorical	No *, Yes	Health care in preceding y ^a^	Categorical	No *, Yes
Financial standard during childhood ^a^	Categorical	Very good/Good *, Average or Poor/Very poor	**Physical performance**		
Unhappy childhood ^a^	Categorical	No *, Yes	Grip strength ^c^	Continuous	Kilogram
Parent having alcohol problem ^a^	Categorical	No *, Yes	Gait speed ^c^	Continuous	meter/second
Being religious ^a^	Categorical	No *, Yes/Undecided	ECG abnormalities ^c^	Categorical	1 = No, 2 = Yes
Smoking status ^a^	Categorical	Never-smoker *, Ever-smoker (i.e., past and current)	FEV1/FVC ratio (FEV%) ^c^	Continuous	Volume first second/Liters
Physically active ^a^	Categorical	Never, ≥1 times/month *	Body Mass Index (unhealthy weight) ^c^	Continuous	No, BMI ≤ 30, Yes, BMI ≥ 31
Overall satisfaction with sleep ^a^	Categorical	Dissatisfied, Satisfied *	**Mental health**		
Alcohol-related injuries to others ^a^	Categorical	No *, Yes	Mini Mental State Examination score ^c^	Continuous	0 to 30 (maximum score)
Others concern about drinking ^a^	Categorical	No *, Yes	Mental Component Summary score ^e^	Continuous	0 (Poor) to 100 (Highest)
**Physical health**			Montgomery Åsberg Depression Rating score ^e^	Continuous	0 to 60 (greater symptom burden)
Life satisfaction ^a^	Continuous	1 (Completely satisfied) to 7 (Dissatisfied)	Brief Scale of Anxiety ^e^	Continuous	0 to 60 (greater symptom burden)
General self-rated health ^a^	Continuous	1 (Excellent) to 5 (Poor)	Minor depression ^f^	Categorical	No *, Yes
Physical Component Summary score ^e^	Continuous	0 (Poor) to 100 (Highest)	Major depression ^f^	Categorical	No *, Yes
Activities of Daily Living score ^d^	Continuous	0 (Lowest) to 8 (Highest)	**Personality traits**		
Instrumental Activities of Daily Living score ^d^	Continuous	0 (Lowest) to 8 (Highest)	Neuroticism score ^e^	Continuous	0 to 24 (maximum score)
CIRS-G score ^d^	Continuous	0 (Lowest) to 56 (Highest)	Extroversion score ^e^	Continuous	0 to 24 (maximum score)

CIRS-G, the Cumulative Illness Rating Scale for Geriatrics; FEV1/FVC, forced expiratory volume in one second divided by forced vital capacity. ^a^ Self-report (interview or questionnaire); ^b^ National registration; ^c^ Test/examination; ^d^ Rating by research nurse; ^e^ Summary score; ^f^ Diagnosed by computerized symptom algorithms. * Reference in logistic regression analyses.

**Table 3:** Sample characteristics, 70-year-olds born in 1944 (examined 2014–16) stratified by sex (*n* = 1156).

	All	Men	Women	Sex Difference
% (no. of cases/total sample)	100.0 (1156)	46.6 (539/1156)	53.4 (617/1156)	
				*p*-value ^a^
Education				0.012 *
Primary education	14.3 (165/1154)	16.7 (90/539)	12.2 (75/615)	
Secondary education	39.4 (455/1154)	28.8 (155/539)	48.8 (300/615)	
Higher education	46.3 (534/1154)	54.5 (294/539)	39.0 (240/615)	
Monthly income SEK, median (min, max)	14,000 (1300, 100,000)	16,915 (3795, 100,000)	12,000 (1300, 50,000)	<0.001 *
Employed	21.6 (248/1147)	23.4 (125/534)	20.1 (123/613)	0.170
Born in Sweden	85.3 (983/1153)	83.3 (449/539)	87.0 (534/614)	0.080
Special housing	1.4 (16/1145)	1.3 (7/538)	1.5 (9/607)	0.794
Having partner	73.3 (846/1154)	83.7 (451/539)	64.2 (395/615)	<0.001 *
Living alone	36.0 (416/1154)	27.6 (149/539)	43.4 (267/615)	<0.001 *
Being religious	24.7 (273/1104)	20.9 (107/511)	28.0 (166/593)	<0.001 *
Smoking				0.053
Never-smoker	38.1 (440/1155)	37.0 (199/538)	39.1 (241/617)	
Current smoker	8.9 (103/1155)	7.1 (38/538)	10.5 (65/617)	
Past smoker	53.0 (612/1155)	55.9 (301/538)	50.4 (311/617)	
Alcohol consumption level				<0.001 *
Lifetime abstainer	2.8 (32/1156)	1.9 (10/539)	3.6 (22/617)	
Former drinker	2.8 (32/1156)	2.6 (14/539)	2.9 (18/617)	
Moderate drinker (≤98 g/week)	64.4 (745/1156)	52.5 (283/539)	74.9 (462/617)	
At-risk drinker (>98 g/week)	30.0 (347/1156)	43.0 (232/539)	18.6 (115/617)	

^a^ Pearson’s chi-square for categorical variables. Mann–Whitney U test for medians. * *p* < 0.05.

**Table 4:** Sociodemographic, social, and health-related factors associated with lifetime abstention, former drinking and at-risk consumption.

	Moderate Consumption ^a^	Lifetime Abstention			Former Drinking			At-Risk Consumption ^b^		
Variables		Adjusted OR	95% CI	*p*	Adjusted OR	95% CI	*p*	Adjusted OR	95% CI	*p*
Secondary education	1.00 (reference)	1.14	0.40–3.22	0.801	0.64	0.26–1.61	0.344	1.58	1.01–2.46	0.045 *
Higher education	1.00 (reference)	0.79	0.27–2.33	0.670	0.49	0.19–1.26	0.139	1.99	1.30–3.04	0.002 *
Income < median	1.00 (reference)	2.86	1.14–7.17	0.026 *	4.33	1.33–14.12	0.015 *	0.57	0.42–0.78	0.001 *
Born outside Sweden	1.00 (reference)	2.16	0.97–4.81	0.059	3.75	1.78–7.90	0.001 *	0.42	0.27–0.65	<0.001 *
Having partner	1.00 (reference)	0.99	0.46–2.16	0.982	0.50	0.24–1.05	0.066	1.48	1.06–2.05	0.021 *
Lost partner >5 years	1.00 (reference)	0.65	0.29–1.45	0.292	2.09	1.00–4.40	0.051	1.46	1.11–1.93	0.008 *
Lost relatives or friends preceding 5 years	1.00 (reference)	0.35	0.14–0.85	0.021 *	1.07	0.40–2.88	0.892	0.97	0.70–1.35	0.872
Alcohol-related injuries to others	1.00 (reference)	n.a.			12.66	2.30–69.83	0.004 *	2.52	0.75–8.39	0.133
Unhappy childhood	1.00 (reference)	0.62	0.19–2.08	0.438	3.61	1.67–7.84	0.001 *	1.24	0.85–1.83	0.268
Being religious	1.00 (reference)	4.19	1.99–8.79	<0.001 *	2.13	0.95–4.79	0.066	0.47	0.33–0.67	<0.001 *
Ever-smoker	1.00 (reference)	0.10	0.04–0.29	<0.001 *	3.79	1.44–9.94	0.007 *	1.76	1.32–2.33	<0.001 *
Physically inactive	1.00 (reference)	2.50	1.19–5.26	0.016 *	2.36	1.08–5.19	0.032 *	0.86	0.67–1.13	0.291
Dissatisfied with sleep	1.00 (reference)	0.73	0.17–3.11	0.666	4.08	1.73–9.59	0.001 *	0.77	0.46–1.30	0.332
Others concern about drinking	1.00 (reference)	n.a.			3.39	0.73–15.70	0.118	4.48	2.39–8.39	<0.001 *
Poor financial standard during childhood	1.00 (reference)	1.38	0.61–3.13	0.435	1.65	0.70–3.86	0.251	0.65	0.50–0.86	0.003 *
Life satisfaction	1.00 (reference)	1.08	0.82–1.42	0.566	1.39	1.10–1.76	0.005 *	0.89	0.79–1.00	0.051
General self-rated health	1.00 (reference)	1.20	0.82–1.77	0.347	1.65	1.08–2.51	0.020 *	0.92	0.79–1.06	0.238
Physical Component Summary score	1.00 (reference)	0.99	0.95–1.02	0.408	0.94	0.91–0.97	<0.001 *	1.00	0.99–1.02	0.850
CIRS-G score	1.00 (reference)	1.02	0.93–1.11	0.715	1.16	1.07–1.27	<0.001 *	1.02	0.99–1.06	0.205
Medications	1.00 (reference)	0.97	0.88–1.08	0.624	1.18	1.09–1.27	<0.001 *	1.01	0.97–1.05	0.751
Hypertension	1.00 (reference)	0.90	0.44–1.83	0.760	2.20	1.05–4.63	0.038 *	1.29	0.99–1.68	0.063
Diabetes	1.00 (reference)	0.84	0.25–2.84	0.784	1.77	0.71–4.44	0.224	0.49	0.30–0.80	0.004 *
Hospitalization preceding 10 years	1.00 (reference)	0.75	0.36–1.55	0.431	4.41	1.78–10.95	0.001 *	1.06	0.81–1.38	0.686
Health care in preceding year	1.00 (reference)	0.27	0.11–0.65	0.004 *	2.23	0.30–16.68	0.436	0.65	0.41–1.04	0.070
Hospital admitted head injury	1.00 (reference)	0.47	0.11–2.00	0.306	3.04	1.39–6.65	0.005 *	1.01	0.97–1.05	0.813
Grip strength	1.00 (reference)	1.01	0.99–1.04	0.353	0.96	0.94–0.98	<0.001 *	1.01	1.00–1.02	0.116
Self-selected gait speed (m/s)	1.00 (reference)	0.85	0.08–9.09	0.896	3.28	1.06–10.19	0.040 *	0.67	0.25–1.81	0.434
Body Mass Index ≥ 31	1.00 (reference)	0.70	0.23–2.64	0.698	2.58	1.12–5.95	0.026 *	1.08	0.72–1.62	0.728
Mini Mental State Examination score	1.00 (reference)	0.95	0.73–1.23	0.672	1.08	0.79–1.47	0.639	1.15	1.03–1.28	0.016 *
Montgomery Åsberg Depression Rating score	1.00 (reference)	1.04	0.98–1.10	0.162	1.06	1.01–1.12	0.032 *	1.02	0.99–1.04	0.249
Minor depression	1.00 (reference)	2.82	1.03–7.75	0.044 *	3.11	1.13–8.56	0.028 *	1.24	0.71–2.17	0.449

Logistic regression models adjusted for sex with moderate consumption as reference category. CIRS-G, Cumulative Illness Rating Scale for Geriatrics. ^a^ Moderate consumption: ≤98 g/week; ^b^ At-risk consumption: >98 g/week. * *p* < 0.05.

**Table 5:** Sociodemographic, social, and health-related factors associated with lower at-risk, medium at-risk, and higher at-risk consumption.

	Moderate Consumption ^a^	Lower At-Risk Consumption ^b^			Medium At-Risk Consumption ^c^			Higher At-Risk Consumption ^d^		
Variables		Adjusted OR	95% CI	*p*	Adjusted OR	95% CI	*p*	Adjusted OR	95% CI	*p*
Secondary education	1.00 (reference)	1.36	(0.82–2.26)	0.240	3.06	(1.23–7.60)	0.016 *	1.06	(0.34–3.31)	0.923
Higher education	1.00 (reference)	1.85	(1.14–2.99)	0.012 *	3.20	(1.33–7.75)	0.010 *	0.87	(0.30–2.57)	0.806
Income below median	1.00 (reference)	0.65	(0.45–0.92)	0.016 *	0.31	(0.16–0.59)	<0.001 *	1.05	(0.42–2.61)	0.919
Born outside Sweden	1.00 (reference)	0.39	(0.23–0.67)	0.001 *	0.25	(0.10–0.63)	0.004 *	1.55	(0.63–3.82)	0.346
Having partner	1.00 (reference)	1.67	(1.13–2.47)	0.010 *	1.02	(0.60–1.74)	00.94	2.06	(0.60–7.10)	0.251
Living alone	1.00 (reference)	0.66	(0.47–0.92)	0.014 *	1.30	(0.82–2.07)	00.26	0.52	(0.19–1.40)	0.195
Lost partner preceding 5 years	1.00 (reference)	0.89	(0.43–1.84)	0.754	1.76	(0.72–4.30)	0.217	16.40	(2.11–127.27)	0.007 *
Lost partner >5 years	1.00 (reference)	1.41	(1.03–1.94)	0.032 *	1.43	(0.89–2.23)	0.136	1.91	(0.86–4.26)	0.113
Poor financial standard during childhood	1.00 (reference)	0.69	(0.50–0.94)	0.019 *	0.67	(0.42–1.05)	0.080	0.44	(0.20–0.98)	0.046 *
Being religious	1.00 (reference)	0.50	(0.33–0.74)	0.001 *	0.44	(0.23–0.83)	0.012 *	0.38	(0.11–1.28)	0.117
Ever-smoker	1.00 (reference)	1.55	(1.13–2.14)	0.007 *	2.29	(1.36–3.84)	0.002 *	2.34	(0.92–5.97)	0.074
Others concern about drinking	1.00 (reference)	3.33	(1.63–6.80)	0.001 *	5.54	(2.43–12.63)	<0.001 *	9.68	(3.23–29.01)	<0.001 *
Life satisfaction	1.00 (reference)	0.82	(0.71–0.95)	0.009 *	1.05	(0.88–1.25)	0.577	0.85	(0.59–1.24)	0.398
CIRS-G score	1.00 (reference)	0.99	(0.85–1.03)	0.721	1.07	(1.01–1.13)	0.018 *	1.10	(1.00–1.21)	0.043 *
Myocardial infarction	1.00 (reference)	0.28	(0.10–0.79)	0.016 *	1.45	(0.66–3.19)	0.351	1.04	(0.23–4.67)	0.957
Diabetes	1.00 (reference)	0.48	(0.27–0.86)	0.013 *	0.45	(0.19–1.08)	0.075	0.80	(0.23–2.76)	0.722
Liver disease	1.00 (reference)	0.17	(0.02–1.31)	0.089	1.346	(0.38–4.79)	0.646	11.41	(3.48–37.37)	<0.001 *
Healthcare in preceding year	1.00 (reference)	0.69	(0.40–1.17)	0.167	0.68	(0.31–1.46)	0.318	0.32	(0.11–0.92)	0.035 *
Grip strength	1.00 (reference)	1.01	(1.00–1.02)	0.011 *	0.99	(0.98–1.01)	0.290	1.01	(0.98–1.03)	0.738
Mini Mental State Examination score	1.00 (reference)	1.18	(1.03–1.34)	0.015 *	1.17	(0.96–1.42)	0.119	0.89	(0.69–1.15)	0.365
Minor depression	1.00 (reference)	0.84	(0.41–1.73)	0.638	1.77	(0.75–4.21)	0.196	4.57	(1.40–14.95)	0.012 *

Logistic regression models adjusted for sex with moderate consumption as reference category. CIRS-G, Cumulative Illness Rating Scale for Geriatrics. ^a^ Moderate consumption: ≤98 g/week; ^b^ Lower at-risk consumption: >98 to <196 g/week; ^c^ Medium at-risk consumption: ≥196 to <350 g/week; ^d^ Higher at-risk consumption: ≥350 g/week. * *p* < 0.05.

**Table 6:** Sociodemographic, social, and health-related factors associated with former drinking.

	Lifetime Abstention	Former Drinking			At-Risk Consumption ^a^	Former Drinking		
Variables		Adjusted OR	95% CI	*p*		Adjusted OR	95% CI	*p*
Secondary education	1.00 (reference)	0.52	(0.14–2.01)	0.344	1.00 (reference)	0.40	(0.15–1.08)	0.070
Higher education	1.00 (reference)	0.53	(0.12–2.24)	0.384	1.00 (reference)	0.24	(0.09–0.64)	0.005 *
Income below median	1.00 (reference)	1.43	(0.33–6.29)	0.633	1.00 (reference)	7.25	(2.22–23.65)	0.001 *
Born outside Sweden	1.00 (reference)	2.00	(0.68–5.86)	0.205	1.00 (reference)	8.78	(3.79–20.35)	0.000 *
Having partner	1.00 (reference)	0.50	(0.17–1.46)	0.205	1.00 (reference)	0.34	(0.16–0.72)	0.005 *
Living alone	1.00 (reference)	2.35	(0.84–6.58)	0.105	1.00 (reference)	2.45	(1.16–5.15)	0.018 *
Lost partner >5 years	1.00 (reference)	3.22	(1.09–9.51)	0.034 *	1.00 (reference)	1.41	(0.66–3.04)	0.377
Poor financial standard during childhood	1.00 (reference)	1.35	(0.41–4.46)	0.621	1.00 (reference)	2.51	(1.05–6.01)	0.038 *
Unhappy childhood	1.00 (reference)	6.20	(1.47–26.13)	0.013 *	1.00 (reference)	2.83	(1.26–6.39)	0.012 *
Being religious	1.00 (reference)	0.55	(0.18–1.64)	0.282	1.00 (reference)	4.42	(1.89–10.37)	0.001 *
Ever-smoker	1.00 (reference)	43.43	(9.62–196.12)	<0.001 *	1.00 (reference)	2.11	(0.78–5.66)	0.139
Physically inactive	1.00 (reference)	1.15	(0.37–3.56)	0.805	1.00 (reference)	2.66	(1.19–5.97)	0.017 *
Dissatisfied with sleep	1.00 (reference)	5.42	(1.02–28.82)	0.048 *	1.00 (reference)	4.87	(1.90–12.48)	0.001 *
Life satisfaction	1.00 (reference)	1.31	(0.91–1.90)	0.152	1.00 (reference)	1.56	(1.21–2.01)	0.001 *
General self-rated health	1.00 (reference)	1.38	(0.78–2.42)	0.267	1.00 (reference)	1.79	(1.17–2.75)	0.008 *
Physical Component Summary score	1.00 (reference)	0.95	(0.90–1.00)	0.031 *	1.00 (reference)	0.93	(0.90–0.97)	<0.001 *
Activities of Daily Living score	1.00 (reference)	0.96	(0.88–1.04)	0.323	1.00 (reference)	0.91	(0.85–0.97)	0.004 *
CIRS-G score	1.00 (reference)	1.13	(1.00–1.27)	0.044 *	1.00 (reference)	1.15	(1.05–1.26)	0.004 *
Medications	1.00 (reference)	1.23	(1.06–1.42)	0.005 *	1.00 (reference)	1.22	(1.11–1.34)	0.000 *
Angina pectoris	1.00 (reference)	3.05	(0.30–31.45)	0.350	1.00 (reference)	6.50	(1.43–29.61)	0.015 *
Diabetes	1.00 (reference)	2.33	(0.52–10.40)	0.270	1.00 (reference)	3.56	(1.30–9.76)	0.013 *
Hospitalization preceding 10 y	1.00 (reference)	6.00	(1.92–18.78)	0.002 *	1.00 (reference)	3.80	(1.51–9.60)	0.005 *
Hospital admitted head injury	1.00 (reference)	6.41	(1.22–33.55)	0.028 *	1.00 (reference)	3.08	(1.35–7.05)	0.008 *
Grip strength	1.00 (reference)	0.96	(0.92–0.99)	0.025 *	1.00 (reference)	0.94	(0.91–0.97)	<0.001 *
Self-selected gait speed	1.00 (reference)	2241.05	(5.84–860659.48)	0.011 *	1.00 (reference)	48.14	(4.85–477.34)	0.001 *

Logistic regression models adjusted for sex with lifetime abstention and at-risk consumption as reference categories. CIRS-G, Cumulative Illness Rating Scale for Geriatrics. ^a^ At-risk consumption: >98 g/week. * *p* < 0.05.

## Data Availability

The data analyzed in the present study are available on request from the corresponding author.

## Supplementary Materials

The following supporting information can be downloaded at: https://www.mdpi.com/article/10.3390/ijerph19148248/s1, Table S1: Associated factors of lifetime abstention, former drinking and at-risk consumption. Logistic regression models adjusted for sex (Model 1) and models adjusted for sex and education (Model 2) with moderate consumption (≤98 g/week) as reference category; Table S2: Associated factors of lower at-risk (>98 to <196 g/week), medium at-risk (≥196 to <350 g/week), and higher at-risk consumption (≥350 g/week). Logistic regression models adjusted for sex (Model 1) and models adjusted for sex and education (Model 2) with moderate consumption (≤98 g/week) as reference category; Table S3: Associated factors of former drinking. Logistic regression models adjusted for sex (Model 1) and models adjusted for sex and education (Model 2) with lifetime abstention and at-risk consumption (>98 g/week) as reference categories.
