# Supplementary material for: Patterns of Alcohol Consumption and Associated Factors in a Population-Based Sample of 70-Year-Olds: Data from the Gothenburg H70 Birth Cohort Study 2014–16
Source: Int J Environ Res Public Health. 2022 Jul 6;19(14):8248. doi: 10.3390/ijerph19148248 (PMC9324895; doi:10.3390/ijerph19148248)
Supplement: Supplementary file 1 [file ijerph-19-08248-s001.zip › ijerph-1773608-supplementary.pdf]

Online-Only Supplemental Material

List of included material

1. **Table S1:** Associated factors of lifetime abstention, former drinking and at-risk consumption. Logistic regression models adjusted for sex (Model 1) and models adjusted for sex and education (Model 2) with moderate consumption (≤98 g/wk) as reference category
2. **Table S2**: Associated factors of lower at-risk (>98 to <196 g/wk), medium at-risk (≥196 to <350 g/wk), and higher at-risk consumption (≥350 g/wk). Logistic regression models adjusted for sex (Model 1) and models adjusted for sex and education (Model 2) with moderate consumption (≤98 g/wk) as reference category
3. **Table S3**: Associated factors of former drinking. Logistic regression models adjusted for sex (Model 1) and models adjusted for sex and education (Model 2) with lifetime abstention and at-risk consumption (>98 g/wk) as reference categories.

Supplementary Table S1. Associated factors of lifetime abstention, former drinking and at-risk consumption (>98 g/w). Logistic regression models adjusted for sex (Model 1) and models adjusted for sex and education (Model 2) with moderate consumption (≤98 g/wk) as reference category

	**Lifetime abstention**			**Former drinking**			**At-risk consumption**		
	**Unadjusted**	**Model 1**	**Model 2**	**Unadjusted**	**Model 1**	**Model 2**	**Unadjusted**	**Model 1**	**Model 2**
	OR (95% CI)	OR (95% CI)	OR (95% CI)	OR (95% CI)	OR (95% CI)	OR (95% CI)	OR (95% CI)	OR (95% CI)	OR (95% CI)
Secondary education	1.20 (0.43-3.34)	1.14 (0.40-3.22)		0.61 (0.25-1.50)	0.64 (0.26-1.61)		1.19 (0.78-1.83)	1.58 (1.01-2.46)*	
Higher education	0.79 (0.27-2.32)	0.79 (0.27-2.33)		0.49 (0.19-1.26)	0.49 (0.19-1.26)		1.87 (1.24-2.81)*	1.99 (1.30-3.04)*	
Income below median	2.56 (1.06-6.17)*	2.86 (1.14-7.17)*	2.87 (1.12-7.38)*	3.06 (0.99-9.49)	4.33 (1.33-14.12)*	4.76 (1.42-15.96)*	0.41 (0.31-0.55)*	0.57 (0.42-0.78)*	0.61 (0.44-0.84)*
Employed	1.10 (0.47-2.60)	1.12 (0.47-2.65)	1.14 (0.48-2.71)	0.87 (0.35-2.15)	0.87 (0.35-2.14)	0.89 (0.36-2.22)	1.14 (0.84-1.55)	1.08 (0.79-1.48)	1.05 (0.76-1.45)
Born outside Sweden	2.07 (0.94-4.59)	2.16 (0.97-4.81)	2.14 (0.96-4.76)	3.83 (1.83-8.02)*	3.75 (1.78-7.90)*	3.76 (1.78-7.92)*	0.50 (0.33-0.77)*	0.42 (0.27-0.65)*	0.42 (0.27-0.65)*
Special housing	n.a.	n.a.	n.a.	n.a.	n.a.	n.a.	0.30 (0.07-1.34)	0.30 (0.07-1.34)	0.29 (0.06-1.34)
Having partner	0.93 (0.43-2.00)	0.99 (0.46-2.16)	0.99 (0.45-2.16)	0.54 (0.27-1.11)	0.50 (0.24-1.05)	0.53 (0.25-1.11)	1.91 (1.39-2.62)*	1.48 (1.06-2.05)*	1.42 (1.02-1.98)*
Happy relationship	1.14 (0.47-2.76)	1.15 (0.47-2.77)	1.20 (0.49-2.91)	2.06 (0.71-5.93)	2.03 (0.70-5.85)	2.05 (0.71-5.95)	0.98 (0.73-1.32)	0.96 (0.71-1.31)	0.94 (0.69-1.27)
Living alone	0.83 (040-1.75)	0.79 (0.37-1.68)	0.79 (0.37-1.69)	1.80 (0.89-3.66)	1.90 (0.93-3.91)	1.82 (0.88-3.75)	0.65 (0.47-0.86)*	0.78 (0.59-1.04)	0.81 (0.61-1.08)
Having children	0.24 (0.03-1.74)	0.23 (0.03-1.71)	0.22 (0.03-1.67)	0.24 (0.03-1.74)	0.24 (0.03-1.76)	0.22 (0.03-1.67)	1.08 (0.74-1.59)	1.12 (0.75-1.67)	1.13 (0.75-1.68)
Having grandchildren	0.92 (0.37-2.28)	0.91 (0.37-2.27)	0.89 (0.36-2.22)	0.85 (0.34-2.09)	0.85 (0.34-2.10)	0.80 (0.32-2.00)	0.92 (0.67-1.27)	0.93 (0.67-1.30)	0.96 (0.69-1.34)
Lost partner preceding 5 y	0.90 (0.21-3.97)	0.86 (0.19-3.78)	0.86 (0.20-3.82)	1.46 (0.32-6.66)	1.54 (0.34-7.10)	1.51 (0.33-6.99)	0.89 (0.50-1.60)	1.06 (0.58-1.95)	1.07 (0.58-1.96)
Lost partner >5 y	0.67 (0.30-1.48)	0.65 (0.29-1.45)	0.64 (0.29-1.43)	2.03 (0.97-4.25)	2.09 (1.00-4.40)	2.03 (0.96-4.27)	1.28 (0.98-1.67)	1.46 (1.11-1.93)*	1.47 (1.11-1.94)*
Lost relatives preceding 5 y	0.37 (0.15-0.90)*	0.35 (0.14-0.85)*	0.35 (0.14-0.85)*	1.07 (0.40-2.88)	1.07 (0.40-2.88)	1.05 (0.39-2.81)	0.92 (0.67-1.25)	0.97 (0.70-1.35)	1.00 (0.72-1.39)
Lost relatives >5 y	0.67 (0.29-1.56)	0.66 (0.29-1.54)	0.64 (0.27-1.49)	1.59 (0.59-4.26)	1.61 (0.60-4.32)	1.54 (0.57-4.14)	0.71 (0.50-1.01)	0.73 (0.51-1.04)	0.75 (0.52-1.09)
Having ≥1 confidant	0.81 (0.19-3.49)	0.86 (0.20-3.77)	0.87 (0.20-3.82)	1.54 (0.45-5.27)	1.33 (0.38-4.67)	1.31 (0.37-4.62)	0.82 (0.49-1.36)	0.60 (0.35-1.01)	0.62 (0.36-1.06)
Feeling alone	0.95 (0.36-2.51)	0.94 (0.36-2.50)	0.97 (0.36-2.57)	1.18 (0.48-2.93)	1.19 (0.48-2.95)	1.17 (0.47-2.91)	0.94 (0.66-1.34)	0.98 (0.68-1.41)	1.00 (0.69-1.45)
Poor financial standard during childhood	1.38 (0.61-3.12)	1.38 (0.61-3.13)	1.34 (0.59-3.06)	1.65 (0.70-3.86)	1.65 (0.70-3.86)	1.55 (0.66-3.67)	0.67 (0.52-0.88)*	0.65 (0.50-0.86)*	0.68 (0.52-0.90)*
Unhappy childhood	0.64 (0.19-2.14)	0.62 (0.19-2.08)	0.62 (0.19-2.08)	3.41 (1.59-7.33)*	3.61 (1.67-7.84)*	3.62 (1.66-7.87)*	1.04 (0.72-1.50)	1.24 (0.85-1.83)	1.25 (0.85-1.84)
Parent having alcohol problem	1.03 (0.42-2.55)	1.03 (0.42-2.55)	1.03 (0.42-2.56)	0.82 (0.31-2.18)	0.82 (0.31-2.18)	0.82 (0.31-2.19)	0.98 (0.70-1.37)	0.93 (0.66-1.31)	0.92 (0.65-1.30)
Being religious	4.21 (2.01-8.84)*	4.19 (1.99-8.79)*	4.34 (2.06-9.15)*	2.09 (0.93-4.68)	2.13 (0.95-4.79)	2.20 (0.97-4.95)	0.45 (0.32-0.64)*	0.47 (0.33-0.67)*	0.46 (0.32-0.66)*

Ever-smoker	0.10 (0.04-0.29)*	0.10 (0.04-0.29)*	0.10 (0.03-0.29)*	3.79 (1.44-9.94)*	3.79 (1.44-9.94)*	3.73 (1.42-9.80)*	1.72 (1.31-2.27)*	1.76 (1.32-2.33)*	1.82 (1.37-2.43)*
Physically inactive	2.51 (1.19-5.28)*	2.50 (1.19-5.26)*	2.52 (1.19-5.32)*	2.36 (1.08-5.19)*	2.36 (1.08-5.19)*	2.26 (1.02-5.00)*	0.85 (0.65-1.11)	0.86 (0.67-1.13)	0.90 (0.69-1.19)
Dissatisfied with sleep	0.72 (0.17-3.07)	0.73 (0.17-3.11)	0.72 (0.17-3.10)	4.10 (1.75-9.63)*	4.08 (1.73-9.59)*	3.97 (1.68-9.38)*	0.73 (0.44-1.21)	0.77 (0.46-1.30)	0.79 (0.47-1.34)
Alcohol-related injuries to others	n.a.	n.a	n.a	11.75 (2.17-63.65)*	12.66 (2.30-69.83)*	12.68 (2.28-70.46)*	2.97 (0.94-9.44)	2.52 (0.75-8.39)	2.39 (0.72-7.93)
Others concern about drinking	n.a.	n.a	n.a	3.62 (0.79-16.61)	3.39 (0.73-15.70)	3.58 (0.77-16.74)	5.15 (2.82-9.43)*	4.48 (2.39-8.39)*	4.23 (2.25-7.93)*
Life satisfaction	1.08 (0.82-1.42)	1.08 (0.82-1.42)	1.08 (0.82-1.42)	1.40 (1.11-1.77)*	1.39 (1.10-1.76)*	1.39 (1.10-1.76)*	0.90 (0.81-1.01)	0.89 (0.79-1.00)	0.90 (0.80-1.02)
General self-rated health	1.21 (0.82-1.78)	1.20 (0.82-1.77)	1.20 (0.81-1.77)	1.62 (1.07-2.47)*	1.65 (1.08-2.51)*	1.64 (1.08-2.50)*	0.89 (0.77-1.03)	0.92 (0.79-1.06)	0.93 (0.80-1.08)
Physical Component Summary score	0.99 (0.95-1.02)	0.99 (0.95-1.02)	0.99 (0.95-1.02)	0.94 (0.91-0.97)*	0.94 (0.91-0.97)*	0.94 (0.91-0.97)*	1.01 (0.99-1.02)	1.00 (0.99-1.02)	1.00 (0.99-1.01)
Activities of Daily Living score	0.98 (0.94-1.03)	0.98 (0.94-1.02)	0.98 (0.94-1.03)	0.97 (0.94-1.00)	0.97 (0.94-1.00)	0.97 (0.94-1.00)	1.02 (0.99-1.05)	1.02 (0.99-1.05)	1.02 (0.99-1.05)
Instrumental Activities of Daily Living score	1.10 (0.49-2.47)	1.09 (0.48-2.46)	1.10 (0.48-2.53)	0.97 (0.53-1.77)	0.98 (0.54-1.79)	1.02 (0.56-1.86)	1.45 (0.92-2.27)	1.57 (0.98-2.51)	1.51 (0.94-2.41)
Cumulative Illness Rating Scale for Geriatrics score	1.02 (0.93-1.11)	1.02 (0.93-1.11)	1.02 (0.93-1.11)	1.16 (1.07-1.26)*	1.16 (1.07-1.27)*	1.16 (1.07-1.27)*	1.02 (0.99-1.06)	1.02 (0.99-1.06)	1.02 (0.99-1.06)
Medications	0.98 (0.88-1.09)	0.97 (0.88-1.08)	0.98 (0.88-1.09)	1.17 (1.09-1.27)*	1.18 (1.09-1.27)*	1.19 (1.10-1.28)*	0.99 (0.95-1.03)	1.01 (0.97-1.05)	1.01 (0.97-1.05)
Stroke	n.a.	n.a.	n.a.	1.53 (0.45-5.21)	1.53 (0.45-5.21)	1.49 (0.44-5.10)	0.72 (0.40-1.29)	0.74 (0.41-1.36)	0.76 (0.41-1.38)
Hypertension	0.90 (0.44-1.83)	0.90 (0.44-1.83)	0.90 (0.44-1.84)	2.20 (1.04-4.62)*	2.20 (1.05-4.63)*	2.17 (1.03-4.57)*	1.31 (0.02-1.70)	1.29 (0.99-1.68)	1.32 (1.01-1.72)
Angina pectoris	0.89 (0.12-6.78)	0.91 (0.12-6.93)	0.96 (1.13-7.31)	2.86 (0.82-9.99)	2.80 (0.80-9.80)	2.90 (0.82-10.25)	0.41 (0.15-1.06)	0.39 (0.15-1.05)	0.40 (1.15-1.08)
Myocardial infarction	1.27 (0.29-5.50)	1.37 (0.31-6.03)	1.35 (0.31-5.95)	1.96 (0.57-6.74)	1.87 (0.54-6.51)	1.72 (0.49-6.03)	0.86 (0.47-1.59)	0.65 (0.35-1.22)	0.68 (0.36-1.29)
Diabetes	0.81 (0.24-2.73)	0.84 (0.25-2.84)	0.88 (0.26-2.97)	1.81 (0.73-4.53)	1.77 (0.71-4.44)	1.79 (0.71-4.53)	0.59 (0.37-0.94)*	0.49 (0.30-0.80)*	0.48 (0.30-0.79)*
Liver disease	1.11 (0.15-8.54)	1.08 (0.14-8.28)	1.15 (0.15-8.89)	2.30 (0.51-10.24)	2.38 (0.53-10.64)	2.56 (0.57-11.56)	0.92 (0.42-2.03)	1.05 (0.46-2.38)	1.05 (0.46-2.38)
Cancer	0.67 (0.24-1.99)	0.69 (0.24-2.01)	0.70 (0.24-2.04)	0.92 (0.35-2.45)	0.91 (0.34-2.43)	0.95 (0.36-2.53)	1.11 (0.80-1.55)	1.06 (0.75-1.50)	1.02 (0.72-1.45)
Hip or femoral fracture preceding 10 y	n.a.	n.a.	n.a.	2.96 (0.36-24.44)	3.00 (0.36-24.81)	3.06 (0.37-25.39)	1.08 (0.32-3.61)	1.31 (0.37-4.59)	1.34 (0.38-4.80)
Have fallen preceding 1 y	1.45 (0.66-3.20)	1.42 (0.64-3.13)	1.47 (0.66-3.26)	0.69 (0.26-1.81)	0.70 (0.27-1.85)	0.70 (0.26-1.86)	0.76 (0.55-1.06)	0.85 (0.61-1.20)	0.87 (0.61-1.22)
Hospital admitted head injury	0.45 (0.11-1.93)	0.47 (0.11-2.00)	0.47 (0.11-2.01)	3.10 (1.42-6.74)*	3.04 (1.39-6.65)*	3.04 (1.39-6.66)*	1.13 (0.78-1.63)	1.01 (0.97-1.05)	1.00 (0.68-1.48)
Home care ≥1/week	n.a.	n.a.	n.a.	n.a.	n.a.	n.a.	0.24 (0.03-1.88)	0.17 (0.02-1.38)	0.16 (0.02-1.31)
Hospitalization preceding 10 y	0.72 (0.35-1.48)	0.75 (0.36-1.55)	0.75 (0.36-.1.55)	4.39 (1.78-10.82)*	4.41 (1.78-10.95)*	4.32 (1.73-10.76)*	1.23 (0.95-1.59)	1.06 (0.81-1.38)	1.07 (0.82-1.40)
Healthcare in preceding y	0.26 (0.11-0.64)*	0.27 (0.11-0.65)*	0.27 (0.11-0.65)*	2.23 (0.30-16.68)	2.23 (0.30-16.68)	2.21 (0.30-16.58)	0.63 (0.40-0.98)*	0.65 (0.41-1.04)	0.64 (0.40-1.02)
Grip strength	1.01 (0.98-1.03)	1.01 (0.99-1.04)	1.01 (0.99-1.04)	0.96 (0.94-0.99)*	0.96 (0.94-0.98)*	0.96 (0.94-0.98)*	1.02 (1.01-1.03)*	1.01 (1.00-1.02)	1.01 (1.00-1.02)

Self-selected gait speed	0.86 (0.08-9.24)	0.85 (0.08-9.09)	0.85 (0.08-8.64)	3.09 (1.00-9.58)	3.28 (1.06-10.19)*	3.15 (1.01-9.88)*	0.65 (0.26-1.66)	0.67 (0.25-1.81)	0.82 (0.31-2.13)
Electrocardiography abnormalities	0.84 (0.40-1.76)	0.88 (0.41-1.87)	0.86 (0.41-1.83)	1.43 (0.65-3.13)	1.39 (0.63-3.06)	1.39 (0.63-3.07)	1.13 (0.86-1.48)	0.95 (0.72-1.26)	0.94 (0.70-1.24)
FEV1/FVC ratio	3.11 (0.16-60.91)	3.14 (0.15-63.85)	2.92 (0.14-60.84)	0.88 (0.04-17.41)	0.89 (0.05-17.35)	0.97 (0.05-19.31)	1.35 (0.48-3.82)	1.30 (0.44-3.80)	1.28 (0.44-3.78)
Body Mass Index ≥31	0.80 (0.24-2.69)	0.79 (0.23-2.64)	0.80 (0.24-2.69)	2.57 (1.12-5.92)*	2.58 (1.12-5.95)*	2.52 (1.09-5.84)*	1.03 (0.70-1.53)	1.08 (0.72-1.62)	1.09 (0.72-1.64)
Mini Mental State Examination score	0.95 (0.73-1.23)	0.95 (0.73-1.23)	0.97 (0.74-1.27)	1.07 (0.79-1.46)	1.08 (0.79-1.47)	1.13 (0.82-1.54)	1.10 (0.99-1.22)	1.15 (1.03-1.28)*	1.11 (0.99-1.24)
Mental Component Summary score	0.99 (0.95-1.03)	0.99 (0.95-1.03)	0.99 (0.95-1.03)	0.98 (0.95-1.02)	0.98 (0.94-1.02)	0.98 (0.94-1.02)	1.00 (0.99-1.02)	0.99 (0.98-1.02)	1.00 (0.98-1.02)
MADRS score	1.04 (0.99-1.10)	1.04 (0.98-1.10)	1.04 (0.98-1.10)	1.06 (1.00-1.12)*	1.06 (1.01-1.12)*	1.06 (1.01-1.12)*	1.01 (1.01-1.12)*	1.02 (0.99-1.04)	1.02 (0.99-1.04)
Brief Scale for Anxiety	1.01 (0.95-1.08)	1.01 (0.95-1.08)	1.01 (0.94-1.07)	1.00 (0.94-1.07)	1.01 (0.94-1.07)	1.01 (0.94-1.07)	1.00 (0.98-1.03)	1.02 (1.00-1.05)	1.02 (1.00-1.05)
Minor depression	2.94 (1.08-8.00)*	2.82 (1.03-7.75)*	2.86 (1.04-7.87)*	2.94 (1.08-8.00)*	3.11 (1.13-8.56)*	3.18 (1.15-8.79)*	1.03 (0.60-1.76)	1.24 (0.71-2.17)	1.24 (0.71-2.17)
Major depression	n.a.	n.a.	n.a.	n.a.	n.a.	n.a.	1.45 (0.65-3.26)	1.73 (0.74-4.04)	1.75 (0.75-4.13)
Neuroticism score	0.93 (0.84-1.03)	0.92 (0.83-1.03)	0.92 (0.83-1.03)	1.07 (0.98-1.17)	1.08 (0.99-1.18)	1.08 (0.99-1.18)	0.98 (0.95-1.02)	1.01 (0.97-1.04)	1.01 (0.97-1.04)
Extroversion score	0.95 (0.86-1.05)	0.95 (0.86-1.05)	0.95 (0.86-1.05)	0.99 (0.89-1.10)	0.99 (0.89-1.10)	0.99 (0.89-1.11)	1.02 (0.98-1.06)	1.02 (0.98-1.06)	1.02 (0.98-1.06)

FEV1/FVC; forced expiratory volume in one second (FEV1) divided by forced vital capacity (FVC); MADRS, Montgomery Åsberg Depression Rating Scale.
*$p<.05$

Supplementary Table S2. Associated factors of lower at-risk (>98 to <196 g/wk), medium at-risk (≥196 to <350 g/wk), and higher at-risk consumption (≥350 g/wk). Logistic regression models adjusted for sex (Model 1) and models adjusted for sex and education (Model 2) with moderate consumption (≤98 g/wk) as reference category

	**Lower at-risk consumption**			**Medium at-risk consumption**			**Higher at-risk consumption**		
	**Unadjusted**	**Model 1**	**Model 2**	**Unadjusted**	**Model 1**	**Model 2**	**Unadjusted**	**Model 1**	**Model 2**
	OR (95% CI)	OR (95% CI)	OR (95% CI)	OR (95% CI)	OR (95% CI)	OR (95% CI)	OR (95% CI)	OR (95% CI)	OR (95% CI)
Secondary education	1.06 (0.65-1.74)	1.35 (0.82-2.26)		2.18 (0.89-5.32)	3.06 (1.23-7.60)*		0.67 (0.22-2.05)	1.06 (0.34-3.31)	
Higher education	1.78 (1.11-2.86)*	1.84 (1.14-2.99)*		3.06 (1.28-7.31)*	3.20 (1.33-7.75)*		0.86 (0.30-2.50)	0.87 (0.30-2.57)	
Income below median	0.49 (0.35-0.69)*	0.65 (0.45-0.92)*	0.68 (0.48-1.00)	0.20 (0.11-0.38)*	0.31 (0.16-0.59)*	0.34 (0.17-0.66)*	0.50 (0.21-1.20)	1.05 (0.42-2.61)	1.03 (0.39-2.67)
Paid employment	1.12 (0.79-1.60)	1.06 (0.74-1.53)	1.03 (0.71-1.48)	1.06 (0.63-1.80)	1.00 (0.58-1.72)	0.97 (0.56-1.68)	1.68 (0.72-3.93)	1.57 (0.66-3.73)	1.60 (0.67-3.82)
Born outside Sweden	0.45 (0.27-0.76)*	0.39 (0.23-0.67)*	0.39 (0.23-0.66)*	0.30 (0.12-0.77)*	0.25 (0.10-0.63)*	0.25 (0.10-0.63)*	1.95 (0.80-4.75)	1.55 (0.63-3.82)	1.56 (0.63-3.86)
Special housing	0.23 (0.03-1.75)	0.23 (0.03-1.78)	0.21 (0.03-1.67)	n.a.	n.a.	n.a.	2.07 (0.26-16.33)	1.86 (0.22-15.47)	1.92 (0.23-16.01)
Having partner	2.06 (1.41-3.01)*	1.67 (1.13-2.47)*	1.61 (1.09-2.39)*	1.43 (0.86-2.39)	1.02 (0.60-1.74)	0.93 (0.54-1.60)	3.25 (0.97-10.92)	2.06 (0.60-7.10)	2.09 (0.60-7.23)
Happy relationship	1.02 (0.73-1.43)	1.00 (0.71-1.41)	0.97 (0.69-1.37)	0.91 (0.55-1.51)	0.86 (0.51-1.45)	0.87 (0.52-1.48)	0.94 (0.41-2.16)	0.86 (0.37-2.01)	0.89 (0.37-2.13)
Living alone	0.56 (0.41-0.78)*	0.66 (0.47-0.92)*	0.68 (0.48-0.95)*	1.02 (0.66-1.59)	1.30 (0.82-2.07)	1.41 (0.88-2.25)	0.38 (0.14-1.01)	0.52 (0.19-1.40)	0.50 (0.18-1.38)
Having children	1.14 (0.74-1.77)	1.16 (0.74-1.81)	1.17 (0.75-1.84)	1.09 (0.57-2.08)	1.21 (0.62-2.35)	1.26 (0.65-2.47)	0.61 (0.14-2.61)	0.67 (0.15-2.93)	0.67 (0.15-2.92)
Having grandchildren	0.92 (0.64-1.34)	0.94 (0.64-1.37)	0.97 (0.66-1.43)	0.98 (0.57-1.68)	1.01 (0.58-1.74)	1.06 (0.61-1.84)	0.70 (0.24-2.07)	0.72 (0.24-2.16)	0.72 (0.24-2.18)
Lost partner preceding 5 y	0.79 (0.39-1.61)	0.89 (0.43-1.84)	0.90 (0.43-1.86)	1.38 (0.59-3.23)	1.76 (0.72-4.29)	1.79 (0.73-4.39)	n.a.	n.s.	n.a.
Lost partner >5 y	1.28 (0.94-1.74)	1.41 (1.03-1.94)*	1.43 (1.04-1.96)*	1.20 (0.76-1.89)	1.43 (0.89.2.29)	1.47 (0.91-2.36)	1.56 (0.71-3.40)	1.91 (0.86-4.26)	1.89 (0.85-4.23)
Lost relatives preceding 5 y	0.89 (0.62-1.29)	0.97 (0.67-1.42)	0.99 (0.68-1.44)	0.99 (0.60-1.64)	1.08 (0.64-1.81)	1.12 (0.67-1.90)	0.79 (0.26-2.46)	0.95 (0.30-3.00)	0.93 (0.29-2.95)
Lost relatives >5 y	0.75 (0.47-1.12)	0.78 (0.51-1.18)	0.80 (0.53-1.21)	0.40 (0.20-0.78)*	0.41 (0.21-0.81)*	0.43 (0.22-0.86)*	1.91 (0.67-5.45)	2.08 (0.72-6.04)	2.09 (0.72-6.08)
Having ≥ 1 confidant	0.78 (0.43-1.43)	0.59 (0.32-1.10)	0.61 (0.33-1.14)	0.86 (0.36-2.06)	0.59 (0.24-1.44)	0.63 (0.26-1.55)	0.98 (0.23-4.26)	0.60 (0.14-2.64)	0.60 (0.14-2.66)
Feeling alone	0.94 (0.63-1.42)	0.98 (0.65-1.49)	0.99 (0.65-1.52)	0.93 (0.51-1.70)	0.97 (0.52-1.79)	1.03 (0.55-1.92)	0.93 (0.32-2.75)	0.96 (0.32-2.89)	0.97 (0.32-2.92)
Poor financial standard during childhood	0.71 (0.52-0.97)	0.68 (0.50-0.94)	0.72 (0.52-0.99)	0.66 (0.42-1.02)	0.67 (0.42-1.05)	0.72 (0.46-1.15)	0.46 (0.21-1.01)	0.44 (0.20-0.98)*	0.43 (0.19-0.97)*
Unhappy childhood	1.23 (0.81-1.85)	1.44 (0.94-2.20)	1.43 (0.93-2.18)	0.60 (0.28-1.27)	0.71 (0.33-1.53)	0.71 (0.33-1.55)	1.13 (0.38-3.34)	1.47 (0.48-4.47)	1.46 (0.48-4.45)
Parent having alcohol problem	0.69 (0.62-1.37)	0.90 (0.60-1.34)	0.88 (0.59-1.32)	1.02 (0.59-1.79)	1.01 (0.57-1.79)	1.01 (0.57-1.80)	1.41 (0.55-3.59)	1.39 (0.54-3.61)	1.41 (0.54-3.65)
Being religious	0.48 (0.32-0.71)*	0.50 (0.33-0.74)*	0.48 (0.32-0.73)*	0.42 (0.22-0.78)*	0.44 (0.23-0.83)*	0.43 (0.22-0.81)*	0.35 (0.10-1.17)	0.38 (0.11-1.28)	0.38 (0.11-1.29)

Ever-smoker	1.50 (0.09-2.05)	1.55 (1.13-2.14)*	1.59 (1.16-2.20)*	2.34 (1.40-3.89)*	2.29 (1.36-3.84)*	2.35 (1.40-3.97)*	2.33 (0.93-5.89)	2.34 (0.92-5.97)	2.33 (0.91-5.93)
Physically inactive	0.73 (0.54-0.99)*	0.75 (0.54-1.02)	0.77 (0.56-1.06)	1.26 (0.81-1.95)	1.26 (0.80-1.97)	1.33 (0.85-2.09)	0.74 (0.32-1.69)	0.73 (0.31-1.69)	0.72 (0.31-1.68)
Dissatisfied with sleep	0.65 (0.35-1.20)	0.67 (0.36-1.25)	0.68 (0.36-1.28)	0.75 (0.32-1.79)	0.74 (0.31-1.79)	0.79 (0.32-1.93)	1.40 (0.41.4.81)	1.34 (0.38-4.68)	1.36 (0.39-4.79)
Alcohol-related injuries to others	3.22 (0.92-11.22)	2.87 (0.79-10.42)	2.78 (0.77-10.04)	3.24 (0.62-16.96)	2.77 (0.49-15.64)	2.51 (0.45-14.11)	n.a.	n.a.	n.a.
Others concern about drinking	3.79 (1.90-7.57)*	3.33 (1.63-6.80)*	3.17 (1.55-6.49)*	6.76 (3.08-14.82)*	5.54 (2.43-12.63)*	5.11 (2.22-11.75)*	13.01 (4.61-36.75)*	9.68 (3.23-29.01)*	10.00 (3.20-30.26)*
Life satisfaction	0.83 (0.72-0.95)*	0.82 (0.71-0.95)*	0.83 (0.72-0.96)*	1.07 (0.91-1.27)	1.05 (0.88-1.25)	1.08 (0.90-1.28)	0.87 (0.61-1.25)	0.85 (0.59-1.24)	0.85 (0.58-1.23)
General self-rated health	0.87 (0.74-1.03)	0.90 (0.76-1.07)	0.91 (0.77-1.08)	0.95 (0.74-1.20)	0.97 (0.77-1.24)	1.00 (0.78-1.28)	0.83 (0.54-1.28)	0.89 (0.57-1.37)	0.88 (0.57-1.37)
Physical Component Summary score	1.01 (0.99-1.03)	1.00 (0.99-1.02)	1.00 (0.99-1.02)	1.00 (0.98-1.02)	0.99 (0.97-1.02)	0.99 (0.97-1.01)	1.01 (0.97-1.05)	1.00 (0.96-1.05)	1.00 (0.96-1.05)
Activities of Daily Living score	1.01 (0.98-1.04)	1.01 (0.98-1.04)	1.01 (0.98-1.04)	1.05 (0.98-1.12)	1.05 (0.97-1.12)	1.04 (0.97-1.12)	1.02 (0.93-1.14)	1.03 (0.93-1.13)	1.03 (0.93-1.13)
Instrumental Activities of Daily Living score	1.95 (0.88-4.34)	2.05 (0.90-4.66)	1.94 (0.86-4.35)	1.03 (0.67-1.60)	1.11 (0.72-1.73)	1.05 (0.67-1.66)	n.a.	n.a.	n.a.
Cumulative Illness Rating Scale for Geriatrics score	0.99 (0.95-1.03)	0.99 (0.95-1.03)	0.99 (0.95-1.03)	1.07 (1.01-1.13)*	1.07 (1.01-1.13)*	1.07 (1.02-1.14)*	1.10 (1.01-1.20)*	1.10 (1.01-1.21)*	1.10 (0.01-1.21)*
Medications	0.98 (0.93-1.02)	0.99 (0.95-1.04)	0.99 (0.95-1.04)	1.01 (0.95-1.07)	1.03 (0.97-1.09)	1.03 (0.97-1.10)	1.02 (0.92-1.13)	1.05 (0.94-1.17)	1.06 (0.94-1.18)
Stroke	0.47 (0.21-1.06)	0.49 (0.22-1.12)	0.50 (0.22-1.14)	1.22 (0.53-2.78)	1.21 (0.52-2.84)	1.28 (0.54-3.01)	1.23 (0.28-5.37)	1.21 (0.27-5.39)	1.20 (0.27-5.43)
Hypertension	1.24 (0.93-1.67)	1.25 (0.92-1.69)	1.27 (0.94-1.72)	1.31 (0.85-2.02)	1.28 (0.82-1.99)	1.32 (0.84-2.07)	2.17 (0.96-4.94)	2.22 (0.97-5.10)	2.21 (0.96-5.09)
Angina pectoris	0.37 (0.11-1.22)	0.36 (0.11-1.23)	0.37 (0.11-1.26)	0.62 (0.15-2.66)	0.54 (0.12-2.37)	0.56 (0.13-2.45)	n.a.	n.a.	n.a.
Myocardial infarction	0.34 (0.12-0.96)*	0.28 (0.10-0.79)*	0.28 (0.10-0.81)*	2.06 (0.96-4.41)	1.45 (0.66-3.19)	1.60 (0.72-3.55)	1.58 (0.36-6.95)	1.04 (0.23-4.67)	1.03 (0.23-4.64)
Diabetes	0.55 (0.31-0.98)*	0.48 (0.27-0.86)*	0.46 (0.26-0.83)*	0.55 (0.23-1.29)	0.45 (0.19-1.08)	0.46 (0.19-1.11)	1.03 (0.30-3.49)	0.80 (0.23-2.76)	0.81 (0.23-2.83)
Liver disease	0.15 (0.02-1.13)	0.17 (0.02-1.31)	0.16 (0.02-1.24)	1.16 (0.34-3.97)	1.35 (0.38-4.79)	1.29 (0.36-4.60)	8.20 (2.82-23.84)*	11.41 (3.48-37.37)*	11.40 (3.45-37.63)*
Cancer	1.14 (0.78-1.68)	1.11 (0.75-1.65)	1.07 (0.72-1.59)	0.86 (0.47-1.57)	0.82 (0.44-1.51)	0.78 (0.42-1.44)	1.87 (0.76-4.57)	1.73 (0.70-4.30)	1.75 (0.70-4.37)
Hip or femoral fracture preceding 10 y	1.23 (0.32-4.66)	1.49 (0.38-5.85)	1.58 (0.40-6.28)	1.03 (0.13-8.35)	1.19 (0.14-10.23)	1.10 (0.13-9.61)	n.a.	n.a.	n.a.
Have fallen preceding 1 y	0.79 (0.54-1.16)	0.88 (0.59-1.30)	0.89 (0.60-1.32)	0.72 (0.40-1.29)	0.82 (0.45-1.48)	0.85 (0.47-1.56)	0.67 (0.23-1.99)	0.80 (0.27-2.40)	0.81 (0.27-2.43)
Hospital admitted head injury	0.88 (0.56-1.40)	0.82 (0.51-1.31)	0.81 (0.51-1.30)	1.89 (1.10-3.25)*	1.56 (0.90-2.73)	1.01 (0.93-1.08)	0.89 (0.26-3.02)	0.74 (0.21-2.55)	0.73 (0.21-2.53)
Home care ≥1/week	n.a.	n.a.	n.a.	n.a.	n.a.	n.a.	3.26 (0.40-26.75)	2.05 (0.24-17.49)	2.01 (0.23-17.43)
Hospitalization preceding 10 y	1.20 (0.89-1.62)	1.07 (0.78-1.45)	1.07 (0.79-1.46)	1.50 (0.96-2.32)	1.18 (0.75-1.86)	1.25 (0.79-1.97)	0.77 (0.35-1.70)	0.56 (0.25-1.26)	0.54 (0.24-1.24)
Healthcare in preceding y	0.67 (0.40-1.12)	0.69 (0.40-1.17)	0.67 (0.39-1.13)	0.70 (0.33-1.47)	0.68 (031-1.46)	0.69 (0.32-1.50)	0.32 (0.12-0.89)*	0.32 (0.11-0.92)*	0.31 (0.11-0.90)*
Grip strength	1.02 (0.02-1.03)*	1.01 (1.00-1.02)	1.01 (1.00-1.02)	1.01 (1.00-1.02)	0.99 (0.98-1.01)	0.99 (0.98-1.01)	1.03 (1.00-1.05)*	1.01 (0.98-1.03)	1.01 (0.98-1.03)

Self-selected gait speed	0.63 (0.21-1.90)	0.68 (0.22-2.10)	0.80 (0.27-2.41)	0.91 (0.23-3.64)	0.92 (0.20-4.25)	1.26 (0.32-4.91)	0.16 (0.00-8.87)	0.18 (0.00-8.68)	0.15 (0.00-7.75)
Electrocardiography abnormalities	1.16 (0.85-1.59)	1.02 (0.74-1.41)	1.00 (0.72-1.39)	1.08 (0.68-1.71)	0.85 (0.53-1.37)	0.82 (0.51-1.33)	1.05 (0.46-2.40)	0.76 (0.33-1.77)	0.77 (0.33-1.78)
FEV1/FVC ratio	2.01 (0.61-6.62)	1.91 (0.57-6.45)	1.85 (0.55-6.27)	0.41 (0.06-2.70)	0.46 (0.07-2.97)	0.44 (0.07-2.87)	1.60 (0.06-45.29)	1.52 (0.06-37.85)	1.45 (0.06-36.23)
Body Mass Index ≥31	1.13 (0.72-1.75)	1.16 (0.74-1.83)	1.14 (0.72-1.81)	0.83 (0.40-1.71)	0.83 (0.40-1.75)	0.92 (0.44-1.95)	0.96 (0.28-3.28)	0.99 (0.29-3.42)	0.99 (0.28-3.48)
Mini Mental State Examination score	1.14 (1.01-1.30)*	1.18 (1.03-1.34)*	1.14 (1.00-1.31)	1.11 (0.92-1.34)	1.17 (0.96-1.42)	1.12 (0.91-1.37)	0.85 (0.67-1.09)	0.89 (0.69-1.15)	0.89 (0.68-1.17)
Mental Component Summary score	1.01 (0.99-1.03)	1.01 (0.99-1.03)	1.01 (0.99-1.03)	0.99 (0.96-1.01)	0.98 (0.96-1.01)	0.98 (0.96-1.01)	1.00 (0.96-1.05)	1.00 (0.95-1.05)	1.00 (0.95-1.05)
MADRS score	1.00 (0.97-1.03)	1.00 (0.97-1.04)	1.00 (0.97-1.04)	1.03 (0.99-1.07)	1.04 (1.00-1.08)	1.04 (1.00-1.08)	1.00 (0.93-1.09)	1.01 (0.94-1.10)	1.01 (0.94-1.10)
Brief Scale for Anxiety Score	1.01 (0.98-1.03)	1.02 (0.99-1.05)	1.02 (0.99-1.05)	1.00 (0.96-1.04)	1.02 (0.98-1.06)	1.02 (0.98-1.06)	1.01 (0.94-1.09)	1.04 (0.97-1.11)	1.04 (0.97-1.11)
Minor depression	0.73 (0.36-1.47)	0.84 (0.41-1.73)	0.84 (0.41-1.71)	1.34 (0.58-3.07)	1.77 (0.75-4.21)	1.71 (0.71-4.07)	2.88 (0.95-8.74)	4.57 (1.40-14.95)*	4.53 (1.38-14.87)*
Major depression	1.54 (0.62-3.81)	1.78 (0.70-4.55)	1.82 (0.71-4.68)	1.67 (0.47-5.89)	2.00 (0.54-7.43)	1.91 (0.50-7.27)	n.a.	n.a.	n.a.
Neuroticism score	0.96 (0.93-1.00)	0.99 (0.95-1.03)	0.99 (0.95-1.03)	1.04 (0.98-1.10)	1.06 (1.00-1.11)	1.06 (1.00-1.12)	0.94 (0.84-1.05)	0.97 (0.87-1.08)	0.97 (0.87-1.08)
Extroversion score	1.01 (0.96-1.05)	1.01 (0.96-1.05)	1.01 (0.96-1.05)	1.03 (0.97-1.10)	1.04 (0.97-1.11)	1.04 (0.97-1.10)	1.09 (0.97-1.22)	1.09 (0.97-1.23)	1.09 (0.97-1.23)

FEV1/FVC; forced expiratory volume in one second (FEV1) divided by forced vital capacity (FVC); MADRS, Montgomery Åsberg Depression Rating Scale.
*p<.05

Supplementary Table S3. Associated factors of former drinking. Logistic regression models adjusted for sex (Model 1) and models adjusted for sex and education (Model 2) with lifetime abstention and at-risk consumption (>98 g/wk) as reference categories

	**Lifetime abstention**	**Former drinking**			**At-risk consumption**	**Former drinking**		
		**Unadjusted**	**Model 1**	**Model 2**		**Unadjusted**	**Model 1**	**Model 2**
		OR (95% CI)	OR (95% CI)	OR (95% CI)		OR (95% CI)	OR (95% CI)	OR (95% CI)
Secondary education	1.00	0.51 (0.13-1.93)	0.52 (0.14-2.01)		1.00	0.51 (0.20-1.32)	0.40 (0.15-1.08)	
Higher education	1.00	0.63 (0.16-2.53)	0.53 (0.12-2.24)		1.00	0.27 (0.10-0.70)*	0.24 (0.09-0.64)*	
Income below median	1.00	1.20 (0.29-4.94)	1.43 (0.33-6.29)	1.91 (0.39-9.25)	1.00	7.49 (2.38-23.59)*	7.25 (2.22-23.65)*	7.68 (2.26-26.07)*
Paid employment	1.00	0.79 (0.23-2.69)	0.82 (0.24-2.84)	0.76 (0.22-2.67)	1.00	0.76 (0.30-1.92)	0.78 (0.31-1.99)	0.68 (0.26-1.77)
Born outside Sweden	1.00	1.85 (0.65-5.28)	2.00 (0.68-5.86)	1.98 (0.67-5.81)	1.00	7.63 (3.41-17.04)*	8.78 (3.79-20.35)*	8.00 (3.39-18.88)*
Special housing	1.00	n.a.	n.a.	n.a.	1.00	n.a.	n.a.	n.a.
Having partner	1.00	0.58 (0.21-1.63)	0.50 (0.17-1.46)	0.55 (0.18-1.65)	1.00	0.29 (0.14-0.60)*	0.34 (0.16-0.72)*	0.36 (0.16-0.78)*
Happy relationship	1.00	1.80 (0.46-6.98)	1.85 (0.47-7.24)	1.75 (0.43-7.09)	1.00	2.09 (0.72-6.09)	2.02 (0.69-5.92)	2.10 (0.71-6.21)
Living alone	1.00	2.16 (0.79-5.92)	2.35 (0.84-6.58)	2.21 (0.78-6.29)	1.00	2.76 (1.33-7.74)*	2.45 (1.16-5.15)*	2.41 (1.13-5.16)*
Having children	1.00	1.00 (0.06-16.71)	0.93 (0.05-16.03)	1.06 (0.06-18.53)	1.00	0.22 (0.03-1.63)	0.23 (0.03-1.70)	0.27 (0.04-2.08)
Having grandchildren	1.00	0.92 (0.26-3.26)	0.95 (0.27-3.38)	0.92 (0.26-3.30)	1.00	0.92 (0.36-2.32)	0.93 (0.37-2.38)	0.98 (0.38-2.53)
Lost partner preceding 5 y	1.00	1.62 (0.20-12.91)	1.97 (0.23-16.96)	2.88 (0.29-28.66)	1.00	1.64 (0.34-7.87)	1.43 (0.29-7.02)	1.43 (0.28-7.32)
Lost partner >5 y	1.00	3.05 (1.05-8.84)	3.22 (1.09-9.51)	3.24 (1.09-9.65)	1.00	1.59 (0.75-3.38)	1.41 (0.66-3.04)	1.51 (0.69-3.31)
Lost relatives preceding 5 y	1.00	2.89 (0.79-10.57)	2.73 (0.74-10.12)	2.65 (0.62-11.38)	1.00	1.17 (0.43-3.19)	1.24 (0.45-3.42)	1.08 (0.38-3.06)
Lost relatives >5 y	1.00	2.36 (0.67-8.39)	2.06 (0.55-7.68)	3.41 (0.80-14.56)	1.00	2.24 (0.82-6.16)	2.36 (0.85-6.53)	2.03 (0.71-5.78)
Having ≥ 1 confidant	1.00	1.89 (0.29-12.28)	1.65 (0.25-11.12)	1.59 (0.23-11.17)	1.00	1.88 (0.52-6.75)	2.16 (0.59-7.89)	1.87 (0.49-7.05)
Feeling alone	1.00	1.25 (0.34-4.59)	1.26 (0.34-4.69)	1.12 (0.29-4.33)	1.00	1.26 (0.49-3.20)	1.18 (0.46-3.04)	1.11 (0.43-2.91)
Poor financial standard during childhood	1.00	1.19 (0.37-3.79)	1.35 (0.41-4.46)	1.22 (0.36-4.19)	1.00	2.45 (1.03-5.81)*	2.51 (1.05-6.01)*	2.27 (0.94-5.48)
Unhappy childhood	1.00	5.32 (1.31-21.52)	6.20 (1.47-26.13)	5.81 (1.35-25.07)	1.00	3.28 (1.48-7.26)*	2.83 (1.26-6.39)*	2.66 (1.17-6.07)*
Parent having alcohol problem	1.00	0.80 (0.22-2.95)	0.71 (0.19-2.70)	0.71 (0.18-2.76)	1.00	0.84 (0.31-2.27)	0.99 (0.36-2.72)	1.13 (0.40-3.17)
Being religious	1.00	0.50 (0.17-1.45)	0.55 (0.18-1.64)	0.56 (0.19-1.68)	1.00	4.63 (1.99-10.80)*	4.42 (1.89-10.37)*	4.62 (1.94-11.02)*

Ever-smoker	1.00	37.80 (9.16-155.93)	43.43 (9.62-196.12)	98.53 (11.10-874.29)	1.00	2.20 (0.82-5.86)	2.11 (0.78-5.66)	1.80 (0.66-4.90)
Physically inactive	1.00	0.94 (0.33-2.73)	1.15 (0.37-3.56)	1.10 (0.35-3.49)	1.00	2.78 (1.25-6.21)*	2.66 (1.19-5.97)*	2.29 (1.00-5.25)
Dissatisfied with sleep	1.00	5.71 (1.10-29.66)	5.42 (1.02-28.82)	5.69 (1.05-30.85)	1.00	5.63 (2.24-14-15)*	4.87 (1.90-12.48)*	5.35 (2.03-14.14)*
Alcohol-related injuries to others	1.00	n.a.	n.a.	n.a.	1.00	3.95 (0.78-20.08)	4.07 (0.79-21.02)	3.67 (0.68-19.86)
Others concern about drinking	1.00	n.a.	n.a.	n.a.	1.00	0.70 (0.16-3.09)	0.72 (0.16-3.18)	0.74 (0.17-3.32)
Life satisfaction	1.00	1.32 (0.91-1.89)	1.31 (0.91-1.90)	1.33 (0.91-1.94)	1.00	1.53 (1.19-1.97)*	1.56 (1.21-2.01)*	1.53 (1.19-1.97)*
General self-rated health	1.00	1.33 (0.76-2.31)	1.38 (0.78-2.42)	1.38 (0.78-2.44)	1.00	1.81 (1.18-2.79)*	1.79 (1.17-2.75)*	1.76 (1.14-2.71)*
Physical Component Summary score	1.00	0.95 (0.91-0.99)	0.95 (0.90-0.99)	0.95 (0.90-0.99)	1.00	0.93 (0.90-0.96)*	0.93 (0.90-0.97)*	0.94 (0.91-0.97)*
Activities of Daily Living score	1.00	0.97 (0.89-1.05)	0.96 (0.88-1.04)	0.96 (0.88-1.04)	1.00	0.90 (0.85-0.96)*	0.91 (0.85-0.97)*	0.91 (0.85-0.97)*
Instrumental Activities of Daily Living score	1.00	0.72 (0.15-3.45)	0.59 (0.12-2.97)	0.65 (0.13-3.20)	1.00	0.46 (0.16-1.35)	0.42 (0.14-1.25)	0.46 (0.15-1.42)
Cumulative Illness Rating Scale for Geriatrics score	1.00	1.12 (1.00-1.26)	1.13 (1.00-1.27)	1.13 (1.01-1.28)	1.00	1.16 (1.06-1.27)*	1.15 (1.05-1.26)*	1.14 (1.03-1.25)*
Medications	1.00	1.22 (1.06-1.40)*	1.23 (1.06-1.42)*	1.24 (1.07-1.44)*	1.00	1.23 (1.12-1.35)*	1.22 (1.11-1.34)*	1.21 (1.10-1.33)*
Stroke	1.00	n.a.	n.a.	n.a.	1.00	2.12 (0.58-7.71)	1.91 (0.52-7.07)	2.18 (0.58-8.18)
Hypertension	1.00	2.46 (0.89-6.74)	2.35 (0.85-6.51)	2.39 (0.85-6.69)	1.00	1.67 (0.78-3.57)	1.79 (0.83-3.87)	1.70 (0.78-3.72)
Angina pectoris	1.00	3.21 (0.32-32.60)	3.05 (0.30-31.45)	2.95 (0.28-30.82)	1.00	7.06 (1.61-31.02)*	6.50 (1.43-29.61)*	6.85 (1.41-33.24)*
Myocardial infarction	1.00	1.55 (0.24-9.97)	1.38 (0.21-9.10)	1.21 (0.17-8.79)	1.00	2.28 (0.62-8.35)	2.94 (0.77-11.16)	2.27 (0.57-9.03)
Diabetes	1.00	2.23 (0.51-9.84)	2.33 (0.52-10.40)	2.54 (0.56-11.57)	1.00	3.10 (1.16-8.25)*	3.56 (1.30-9.76)*	3.90 (1.39-10.93)*
Liver disease	1.00	2.07 (0.18-24.01)	2.63 (0.22-31.57)	3.26 (0.26-41.55)	1.00	2.50 (0.52-12.08)	2.11 (0.43-10.50)	1.74 (0.34-8.90)
Cancer	1.00	1.35 (0.33-5.56)	1.30 (0.31-5.44)	1.28 (0.30-5.48)	1.00	0.83 (0.31-2.25)	0.88 (0.32-2.39)	0.92 (0.33-2.53)
Hip or femoral fracture preceding 10 y	1.00	n.a.	n.a.	n.a.	1.00	2.74 (0.30-25.30)	2.67 (0.28-25.70)	2.41 (0.21-27.19)
Have fallen preceding 1 y	1.00	0.47 (0.14-1.61)	0.50 (0.14-1.71)	0.51 (0.15-1.80)	1.00	0.90 (0.33-2.44)	0.83 (0.30-2.26)	0.82 (0.30-2.25)
Hospital admitted head injury	1.00	6.82 (1.36-34.27)*	6.41 (1.22-33.55)*	6.34 (1.20-33.59)*	1.00	2.75 (1.23-6.15)*	3.08 (1.35-7.05)*	2.94 (1.26-6.84)*
Home care ≥1/week	1.00	n.a.	n.a.	n.a.	1.00	n.a.	n.a.	n.a.
Hospitalization preceding 10 y	1.00	6.09 (1.95-18.97)*	6.00 (1.92-18.78)*	7.09 (2.09-24.03)*	1.00	3.56 (1.43-8.90)*	3.80 (1.51-9.60)*	3.76 (1.48-9.55)*
Healthcare in preceding y	1.00	8.46 (0.97-73.64)	8.20 (0.93-71.79)	7.48 (0.83-67.64)	1.00	3.54 (0.47-26.76)	3.08 (0.40-23.54)	3.35 (0.43-25.82)
Grip strength	1.00	0.96 (0.92-0.99)*	0.96 (0.92-0.99)*	0.95 (0.92-0.99)*	1.00	0.94 (0.91-0.97)*	0.94 (0.91-0.97)*	0.95 (0.92-0.97)*

Self-selected gait speed	1.00	882.38 (3.81-204463)*	2241.05 (5.84-860659)*	1628.63 (3.90-680242)*	1.00	43.89 (4.44-433.72)*	48.14 (4.85-477.34)*	33.07 (3.21-340.79)*
Electrocardiography abnormalities	1.00	1.70 (0.59-4.93)	1.60 (0.55-4.69)	1.89 (0.61-5.84)	1.00	1.26 (0.57-2.81)	1.41 (0.63-3.19)	1.52 (0.66-3.46)
FEV1/FVC ratio	1.00	0.26 (0.01-19.19)	0.38 (0.01-31.81)	0.55 (0.01-57.03)	1.00	0.68 (0.04-11.75)	0.71 (0.04-12.46)	0.59 (0.03-12.41)
Body Mass Index ≥31	1.00	3.25 (0.77-13.66)	3.72 (0.85-16.23)	4.36 (0.97-19.68)	1.00	2.48 (1.04-5.91)*	2.30 (0.96-5.53)	2.41 (0.98-5.91)
Mini Mental State Examination score	1.00	1.17 (0.74-1.85)	1.20 (0.76-1.90)	1.16 (0.73-1.86)	1.00	0.97 (0.71-1.33)	0.93 (0.67-1.28)	1.02 (0.74-1.43)
Mental Component Summary score	1.00	1.00 (0.95-1.05)	1.00 (0.94-1.05)	1.00 (0.94-1.05)	1.00	0.98 (0.94-1.02)	0.98 (0.94-1.02)	0.98 (0.94-1.02)
MADRS	1.00	1.02 (0.94-1.09)	1.02 (0.95-1.11)	1.03 (0.95-1.11)	1.00	1.06 (1.00-1.12)	1.04 (0.98-1.11)	1.05 (0.99-1.11)
Brief Scale for Anxiety score	1.00	0.99 (0.91-1.08)	1.01 (0.92-1.10)	1.01 (0.92-1.10)	1.00	1.00 (0.93-1.07)	0.98 (0.91-1.06)	0.99 (0.91-1.06)
Minor depression	1.00	1.00 (0.26-3.86)	1.21 (0.30-4.89)	1.13 (0.27-4.68)	1.00	2.85 (1.00-8.15)	2.51 (0.86-7.32)	2.04 (0.67-6.21)
Major depression	1.00	n.a.	n.a.	n.a.	1.00	n.a.	n.a.	n.a.
Neuroticism score	1.00	1.15 (1.00-1.32)	1.14 (0.99-1.31)	1.14 (0.99-1.31)	1.00	1.09 (1.00-1.20)	1.08 (0.99-1.19)	1.08 (0.99-1.19)
Extroversion score	1.00	1.04 (0.90-1.20)	1.04 (0.90-1.20)	1.04 (0.90-1.21)	1.00	0.97 (0.87-1.09)	0.97 (0.86-1.08)	0.95 (0.85-1.07)

FEV1/FVC; forced expiratory volume in one second (FEV1) divided by forced vital capacity (FVC); MADRS, Montgomery Åsberg Depression Rating Scale.
*p<.05
